# Shell-Sheddable Micelles Based on Poly(ethylene glycol)-hydrazone-poly[R,S]-3-hydroxybutyrate Copolymer Loaded with 8-Hydroxyquinoline Glycoconjugates as a Dual Tumor-Targeting Drug Delivery System

**DOI:** 10.3390/pharmaceutics14020290

**Published:** 2022-01-26

**Authors:** Adrian Domiński, Monika Domińska, Magdalena Skonieczna, Gabriela Pastuch-Gawołek, Piotr Kurcok

**Affiliations:** 1 Centre of Polymer and Carbon Materials, Polish Academy of Sciences, 34, M. Curie-Skłodowskiej St., 41-819 Zabrze, Poland; 2 Department of Organic Chemistry, Bioorganic Chemistry and Biotechnology, Faculty of Chemistry, Silesian University of Technology, Krzywoustego 4, 44-100 Gliwice, Poland; monika.krawczyk@polsl.pl (M.D.); gabriela.pastuch@polsl.pl (G.P.-G.); 3 Biotechnology Centre, Silesian University of Technology, Krzywoustego 8, 44-100 Gliwice, Poland; Magdalena.Skonieczna@polsl.pl; 4 Department of Systems Biology and Engineering, Faculty of Automatic Control, Electronics and Computer Science, Silesian University of Technology, 44-100 Gliwice, Poland

**Keywords:** micelles, pH-responsive, biodegradable, drug release, quinoline glycoconjugates, Warburg effect

## Abstract

The development of selective delivery of anticancer drugs into tumor tissues to avoid systemic toxicity is a crucial challenge in cancer therapy. In this context, we evaluated the efficacy of a combination of nanocarrier pH-sensitivity and glycoconjugation of encapsulated drugs, since both vectors take advantage of the tumor-specific Warburg effect. Herein, we synthesized biodegradable diblock copolymer, a poly(ethylene glycol)-hydrazone linkage-poly[R,S]-3-hydroxybutyrate, which could further self-assemble into micelles with a diameter of ~55 nm. The hydrazone bond was incorporated between two copolymer blocks under an acidic pH, causing the shell-shedding of micelles which results in the drug’s release. The micelles were stable at pH 7.4, but decompose in acidic pH, as stated by DLS studies. The copolymer was used as a nanocarrier for 8-hydroxyquinoline glucose and galactose conjugates as well as doxorubicin, and exhibited pH-dependent drug release behavior. In vitro cytotoxicity, apoptosis, and life cycle assays studies of blank and drug-loaded micelles were performed on Normal Human Dermal Fibroblasts-Neonatal (NHDF-Neo), colon carcinoma (HCT-116), and breast cancer (MCF-7) for 24, 48, and 72 h. A lack of toxicity of blank micelles was demonstrated, whereas the glycoconjugates-loaded micelles revealed enhanced selectivity to inhibit the proliferation of cancer cells. The strategy of combining pH-responsive nanocarriers with glycoconjugation of the drug molecule provides an alternative to the *modus operandi* of designing multi-stimuli nanocarriers to increase the selectivity of anticancer therapy.

## 1. Introduction

Stimuli-responsive polymeric micelles based on amphiphilic well-defined block copolymers have gained great attention over the past decade in the field of anticancer drug delivery [1,2]. Self-assembled nanostructures generally have a hydrophilic outer shell and hydrophobic core. The hydrophilic shell of the micelle prevents steric recognition by the immune system and its removal by the macrophage system from the bloodstream. In contrast, the hydrophobic core can encapsulate fragile hydrophobic anticancer therapeutics [3]. The encapsulation of anticancer drugs in nanocarriers enhances drug accumulation in the tumor tissues through enhanced permeability and the retention effect. Thus, the therapeutic dose of the drug can be reduced, thereby minimizing its side effects [4]. In particular, polymeric nanoparticles derived from biodegradable and biocompatible polyesters such as polylactide [5], poly(ε-caprolactone) [6], poly(3-hydroxybutyrate) [7] or aliphatic polycarbonates [8] are broadly used as drug carriers. Among these, poly(3-hydroxybutyrate) (PHB) is considered to be a future “green” material for biomedical or drug delivery applications [9]. To further its applications in drug delivery systems, it is crucial to obtain PHB in a highly controlled manner with the desired stereochemistry, which is essential for its physicochemical properties [10,11]. Therefore, extensive studies have focused on the ring-opening polymerization of β-lactones to obtain the polymer with the desired molar mass, low dispersity, microstructure, and reduced side reactions [12,13,14,15,16]. The isotactic poly ([R]-3-hydroxybutyrate) is synthesized both biotechnologically through bacterial fermentation and chemically by the polymerization of β-butyrolactone (β-BL) [17]. Both anionic and coordination ring-opening polymerization of β-butyrolactone enable the obtaining of “tailor-made” poly (3-hydroxybutyrate), and its microstructure significantly depends on both the absolute configuration of β-BL and the type of catalyst used [13]. However, the anionic ring-opening polymerization of racemic β-BL—atactic poly([R,S]-3-hydroxybutyrate) (aPHB) is most often synthesized, due to the presence of a chiral C4 carbon in the β-butyrolactone molecule. OligoPHB has been used for drug conjugation to increase metabolic stability, bioavailability, and avoid multi-drug resistance [18,19]. Additionally, poly(3-hydroxybutyrate) is degraded and further resorbs in vivo, meaning that their degradation by-products are eliminated via natural pathways, both during the filtration and after metabolization without causing cytotoxicity. Due to this, PHB has great potential as a building material for anticancer drug delivery systems. However, the non-stimuli-responsive PHB-based micelles have some drawbacks, i.e., the slow drug release from nanocarriers, which significantly lowers the efficiency of therapeutic outcome [20]. Understanding the differences between tumors and healthy tissues is very important when designing new anti-cancer drug carriers. One such difference is the unique metabolism of sugar derivatives by tumor tissues. During the progression, cancer cells significantly increase the glycolysis rate other than mitochondrial oxidative phosphorylation to increase their proliferation [21]. As a consequence, the accumulated pyruvate is mainly converted into lactic acid, which makes the microenvironment of neoplastic tissues slightly acidic (pH 6.5–6.8) [22]. The increased sugar uptake of tumor tissues is related to the overexpression of glucose transporters, which are membrane proteins that facilitate concentration-dependent glucose and galactose uptake inside the cell [23]. This atypical relationship between increased proliferation, the high uptake of sugar derivatives, and the decrease in the extracellular pH of tumor tissues caused by lactate production is known as the Warburg effect [24]. This effect has been found in almost all types of tumors [25]. Therefore, many studies use the Warburg effect in drug delivery applications to improve cancer targeting and selectivity, e.g., by incorporating a sugar unit into the drug structure (glycoconjugation) [26,27] or using pH-sensitive drug carriers [28,29]. Recently, our group reported that a combination of pH-responsive nanocarriers loaded with glycoconjugates significantly improves their selectivity to inhibit the proliferation of cancer cells [30]. In the construction of the pH-responsive nanocarriers, acetal, imine, oxime, vinyl ether, hydrazone or orthoester are typical acid-sensitive chemical linkages, which are stable at physiological pH, but are rapidly hydrolyzed in an acidic environment [31]. The hydrazone linkage has attracted considerable attention in anticancer drug delivery systems, due to its stability at physiological pH and fast hydrolysis in a mildly acidic environment [32]. Moreover, hydrazone-based doxorubicin prodrugs NC-6300 and INNO-206 were reported to be under Phase I and II of clinical trials, respectively [33,34]. In both cases, data revealed that hydrazone-based prodrugs showed significantly higher anticancer activity and reduced side-effects compared to free drugs, an effect which is related to the release of the drug in the tumor tissues. The incorporating acid-labile bonds into the polymer structure, so nanocarriers can be designed to respond in the acidic tumor microenvironment by disassembling, changing their size, shape, surface charge, etc. [35]. As a result, the encapsulated drugs are released in a controlled manner with the ability to target tumor tissues. It is well-known that PEGylation is effective in forming the “stealth” surface of the micelles to avoid non-specific interactions with proteins and prolong the circulation time of nanocarriers in the bloodstream [36]. However, it is also known that PEGylation reduces the cellular uptake of nanoparticles by cancer cells, leading to decreased therapeutic outcomes [37]. Therefore, nanocarriers for cancer treatment should be “stealth” protected with a PEG shell layer, with the capability due to the shell-shedding effect at the tumor site to increase internalization by cancer cells [38]. The unique pathophysiological markers of tumor tissues, i.e., lower pH in the microenvironment of cancer cells, the higher level of reactive oxygen species, high intracellular glutathione levels, hypoxic conditions, or the overexpression of various specific enzymes can act as an endogenous trigger to promote the shell-shedding effect in nanocarriers. As a result, the drug is released in a controlled manner and the ability of the drug to target the tumor tissues is improved. Yang et al. [39] reported a pH-responsive shell-sheddable nanocarrier based on a copolymer of poly(ethylene glycol) and polylactide with an acid-labile acetal linkage bridge for tumor-targeted paclitaxel delivery. The PEG shedding was triggered by tumor’s acidic microenvironment, resulting in prolonged circulation time, enhanced cellular uptake, and an improved in vivo tumor inhibition rate. Manna et al. [40] reported that a four-arm star amphiphilic block copolymer based on [star-(poly(D,L-lactide)-b-poly(N-vinylpyrrolidone)_4_] loaded with methotrexate showed pH-dependent drug release and excellent antitumor efficacy against methotrexate-resistant Dalton lymphoma and Raji cells, whereas a free drug was ineffective. Moreover, the treatment of Dalton lymphoma tumor-bearing mice revealed the prolonged survival of mice with active disease and the generation of CD8+ T-cell-mediated cytolytic responses against the tumor, which was significantly reduced in untreated tumor-bearing mice. Yang et al. [41] prepared dual pH-sensitive polymeric nanoparticles based on a diblock copolymer, consisting of acetal-linked poly(ethylene glycol) and catechol-functionalized aliphatic polycarbonate for anticancer drug delivery. The bortezomib was conjugated to the copolymer through a pH-sensitive boronate ester bond between catechol groups and a drug-derived phenylboronic acid group. The nanocarrier rapidly released bortezomib in response to the acidic tumor microenvironment. Furthermore, the conjugation of paclitaxel via acetal bonding to the poly(ethylene glycol)-b-poly(acrylic acid) copolymer was reported by Zhong and coauthors as a pH-sensitive micellar prodrug with potent antitumor activity against multi-drug-resistant cancer cells [42]. Zhong et al. [43] developed reduction-responsive shell-sheddable micelles from a poly(ethylene glycol)-disulfide linker-poly(ε-caprolactone) copolymer for paclitaxel delivery.

In this work, we report biodegradable copolymer comprising poly(ethylene glycol) and poly([R,S]-3-hydroxybutyrate) blocks linked via hydrazone bond. The resulting amphiphilic copolymer can self-assemble into nanosized shell-sheddable micelles for pH-triggered delivery of encapsulated anticancer agents (8-hydroxyquinoline glycoconjugates and doxorubicin). The hydrazone bond incorporated between two copolymer blocks is broken under the acidic media, causing the shedding of PEG shells, which results in the decomposition of micelles and subsequent drug release. The synthesis of the copolymers, characterization of micelles, pH-dependent degradation of nanocarriers, and drug release profiles were investigated. Moreover, in vitro cytotoxicity of the blank micelles and the activity of drug-loaded mPEG-hyd-aPHB micelles were studied using an MTT (3-[4,5–dimethylthiazol-2-yl]-2,5-diphenyltetrazolium bromide) assay combined with apoptosis and cell cycle assays against healthy cells (NHDF-Neo) and cancer cells (HCT-116 and MCF-7).

## 2. Materials and Methods

### 2.1. Materials

Poly(ethylene glycol) monomethyl ether (mPEG, M_n_ = 5000 g mol^−1^, Sigma-Aldrich, Steinheim, Germany) was dried by azeotropic distillation from anhydrous toluene. Triethylamine (99%, Sigma-Aldrich, Steinheim, Germany), dichloromethane (DCM) (99%, VWR Chemicals, Fontenary-Sous-Bois, France) were dried over CaH_2_ and distilled before use. In addition, 4-Nitrophenyl chloroformate (*p*-NPC 99%, TCI Chemicals, Belgium), hydrazine monohydrate (64–65%, Sigma-Aldrich, Steinheim, Germany) (>98%, Sigma-Aldrich, Steinheim, Germany), levulinic acid (>98%, Alfa Aesar, Haverhill, MA, USA), trifluoroacetic acid (TFA, >99%, Sigma-Aldrich, Steinheim, Germany), pyrene (+98%, Acros Organics, Acros Organics, Geel, Belgium), methanol (MeOH), ethanol, and diethyl ether (all 99%, VWR Chemicals, Fontenary-Sous-Bois, France) were used as received. DMSO (99%, VWR Chemicals, Fontenary-Sous-Bois, France) was distilled under reduced pressure over CaH_2_ and two subsequent reduced pressure distillations over BaO. β-Butyrolactone (>98% Sigma-Aldrich, Steinheim, Germany) was purified as described in [44]. Doxorubicin hydrochloride (DOX·HCl) was purchased from LC Laboratories. The mPEG-COONa was prepared as described in [45]. Model glycoconjugates (see Figure S1) for in vitro cytotoxicity studies, i.e., 8-((1-(2,3,4,6-tetra-*O*-acetyl-β-D-glucopyranosyl)-1H-1,2,3-triazol-4-yl)methoxy)quinoline (8HQ-glucose conjugate–8HQ-Glu) and 8-((1-(2,3,4,6-tetra-*O*-acetyl-β-D-galactopyranosyl)-1H-1,2,3-triazol-4-yl)methoxy)quinoline (8HQ-galactose conjugate—8HQ-Gal) were synthesized as described earlier in [46].

#### Cell Cultures

The Normal Human Dermal Fibroblasts-Neonatal, NHDF-Neo were purchased from LONZA (Cat. No. CC-2509, NHDF-Neo, Dermal Fibroblasts, Neonatal, Lonza, Poland). The human breast adenocarcinoma cell line MCF-7 was obtained from the collection at the Maria Sklodowska-Curie Memorial Cancer Center and National Institute of Oncology (Gliwice, Poland). The human colon adenocarcinoma cell line HCT-116 was obtained from an American Type Culture Collection (ATCC, Manassas, VA, USA). Cells were grown in a humidified atmosphere with 5% CO_2_ and at 37 °C in a culture medium. The culture media consisted of RPMI 1640 (HyClone laboratories, Inc.) or DMEM+F12 (HyClone laboratories, Inc.), supplemented with 10% heat-inactivated fetal bovine serum (FBS) (Eurx, Gdańsk, Poland) and 5% Antibiotic Antimycotic Solution, 100 U/mL penicillin, and 10 mg/mL streptomycin (Sigma-Aldrich, Steinheim, Germany).

### 2.2. Synthesis of mPEG-hyd-LEV Macroinitiator

Under a dry nitrogen atmosphere, the solution of mPEG (5 g, 1.0 mmol, 1.0 eq.) and triethylamine (0.21 mL, 1.5 mmol, 1.5 eq.) in 50 mL of dry DCM was thermostated in 0 °C. Next, a solution of *p*-nitrophenyl chloroformate (*p*-NPC) (2.02 g, 10 mmol, 10 eq.) in 25 mL of dry DCM was introduced dropwise. The reaction mixture was stirred for 30 min at 0 °C. Next, the temperature was increased to room temperature (rt) and the reaction was carried out for 48 h. The DCM was evaporated, excess methanol was added and stirred for 1 h to remove unreacted *p*-NPC. Then, the volatiles were stripped off under reduced pressure, the product was redissolved in DCM, washed three times with brine, and precipitated in cold diethyl ether. The residual solid mPEG-NPC was dried under vacuum to a constant weight. Yield: 85%. ^1^H NMR (600 MHz, CDCl_3_, Figure S2a) δ: 3.38 (3H, CH_3_-O), 3.65 (4mH, CH_2_-CH_2_-O), 4.44 (2H, CH_2_-O-C(O)), 7.4 (2H, Ar H), 8.28 (2H, Ar H). Next, the PEG-NPC (3 g, 0.58 mmol, 1.0 eq.) was dissolved in 50 mL of DCM and slowly added dropwise into hydrazine monohydrate (1.13 mL, 23.23 mmol, ~40 eq.) solution in 50 mL of DCM thermostated at 0 °C. The reaction mixture was stirred for 30 min at 0 °C, and then at rt for 24 h. Then, volatiles were stripped off under vacuum, and the obtained solid was dissolved in deionized water, dialyzed against water (MWCO 1000) at rt for 48 h and lyophilized to obtain mPEG-NH-NH_2_. Yield: 68%. ^1^H NMR (600 MHz, CDCl_3_, Figure S2b) δ: 3.38 (3H, CH_3_-O), 3.65 (4mH, CH_2_-CH_2_-O), 4.27 (2H, CH_2_-O-C(O)). The solution of mPEG-NH-NH_2_ (1 g, 0.2 mmol, 1 eq.) and levulinic acid potassium salt (0.03 g, 0.26 mmol, 1.3 eq.) in 20 mL of dry methanol with 2 drops of TFA was stirred for 48 h at 50 °C. Next, the reaction solution was dialyzed (MWCO 1000) against KOH ethanol solution for 24 h, and against ethanol for 24 h, followed by drying under vacuum at rt to a constant weight. The mPEG-hyd-LEV macroinitiator was stored at −4 °C in glovebox. Yield: 57%. ^1^H NMR (600 MHz, CDCl_3_, Figure S2c) δ: 1.99 (3H, CH_3_-C=N), 2.47 (4H, C-CH_2_-CH_2_-C(O), 3.38 (3H, CH_3_-O), 3.65 (4mH, CH_2_-CH_2_-O), 4.33 (2H, CH_2_-O-C(O)).

### 2.3. Synthesis of mPEG-hyd-aPHB

The polymerizations were performed in the glovebox (H_2_O < 1 ppm, O_2_ < 5 ppm). The ring-opening of β-butyrolactone initiated with hydrazone bond-containing mPEG-hyd-LEV macroinitiator was carried out in dry DMSO at a monomer concentration of 1 mol L^−1^ at rt. Briefly, mPEG-hyd-LEV (0.3 g, 0.05 mmol, 1 eq.) were dissolved in DMSO, after which complete macroinitiator solubilization of the β-butyrolactone (187.9 µL, 2.31 mmol, 40 eq.) was added. Conversion of the monomer was measured by ^1^H NMR spectroscopy, based on the disappearance of the signals derived from the β-butyrolactone. After quantitative monomer conversion, the resulting copolymer was lyophilized and stored at −4 °C in the glovebox. ^1^H NMR (600 MHz, CDCl_3_) δ: 1.28 ppm (3nH, CH_3_), 2.02 (3H, CH_3_-C=N), 2.54 ppm (2nH, CH_2_), 3.38 (3H, CH_3_-O), 3.65 (4mH, CH_2_-CH_2_-O), 4.2 (2H, CH_2_-O-C(O)), 5.25 ppm (1nH, CH). SEC: (DMF, PEG standards): M_n_ = 8200 g mol^−1^, *Đ* = 1.14. 

The mPEG-*b*-aPHB diblock copolymer was synthesized in similar conditions using mPEG-COONa as the macroinitiator. ^1^H NMR (600 MHz, CDCl_3_, Figure S3) δ: 1.28 ppm (3nH, CH_3_), 2.54 ppm (2nH, CH_2_), 3.38 ppm (3H, CH_3_-O), 3.65 ppm (4mH, CH_2_-CH_2_-O), 4.2 ppm (2H, CH_2_-O-C(O)), 5.25 ppm (1nH, C**H**-CH_2_). SEC: (DMF, PEG standards): M_n_ = 7900 g mol^−1^, *Đ* = 1.08.

### 2.4. Characterization of Synthetic Copolymers

The ^1^H NMR spectra were recorded on the Bruker-Avance II 600 MHz with Ultrashield Plus Magnets (Fremont, CA, USA) at room temperature in CDCl_3_ with tetramethylsilane (TMS) as an internal standard. Size exclusion chromatography (SEC) measurements were performed at 45 °C in DMF with the addition of 5 mmol L^−1^ LiBr at a nominal flow rate of 1 mL min^−1^. The chromatography system contained a multiangle light scattering detector (DAWN HELEOS, Wyatt Technology, λ = 658 nm), a refractive index detector (Dn-2010 RI, WGE Dr Bures), and a column system (PSS gel GRAM guard and three columns PSS GRAM 100 Å, 1000 Å and 3000 Å). The molar mass and dispersity were evaluated using ASTRA 5.3.4.10 software from Wyatt Technologies. Poly(ethylene glycol) standards with a low dispersity mass distribution (M_n_ values ranging from 1010 to 29,600 g mol^−1^, Polymer Laboratories) were used to generate a calibration curve. 

### 2.5. Micelles Preparation and Characterization

The solvent evaporation method was used to prepare mPEG-hyd-aPHB and mPEG-b-aPHB micelles. Briefly, 10 mg of the copolymer was dissolved in dry DCM. Next, after the solvent evaporation, a thin film at the bottom of the vial was formed. After removing the traces of organic solvent, the film was dispersed in PBS buffer (10 mL; copolymer concentration was equal to 1 mg · mL^−1^). Next, the mixture was stirred for 60 min at room temperature and sonicated for 30 min in an ice-cold bath. Then, the micelle suspension was filtered using a syringe filter (0.2 μm, Sartorius). The obtained micelle solution was lyophilized and stored at −4 °C in the glovebox. The critical micelle concentration (CMC) was determined using pyrene as a fluorescence probe. A predetermined amount of pyrene (6 × 10^−6^ mol · L^−1^) in acetone was added to micelle solutions of different concentrations (ranging from 1 × 10^−5^ to 1.0 mg · mL^−1^) in phosphate buffer. The samples were incubated overnight at rt in the dark. The fluorescence spectra were recorded on a Hitachi F-2500 Spectrometer (Tokyo, Japan) from 300 to 360 nm with the emission wavelength at 391 nm. The intensity ratio of pyrene at 336 over 333 nm was plotted against the logarithm of copolymer concentration to determine the critical micelle concentration. The hydrodynamic diameter and size distribution of micelles were determined by dynamic light scattering (DLS) measurements using a Zetasizer Nano ZS90 (Malvern Instruments). To examine the stability and acid-triggered degradation of micelles at various pH levels, the 10 mg of freeze-dried micelles was dispersed in 10 mL of PBS buffer with pH 7.4, 6.4, and 5.5 respectively. The mixture was incubated at 37 °C, and at predetermined time intervals, the hydrodynamic diameter of the micelles was measured. The morphologies of the micelles were observed using Cryogenic Transmission Electron Microscopy (cryo-TEM). Images were obtained using a Tecnai F20 X TWIN microscope (FEI Company, Hillsboro, OR, USA) equipped with a field emission gun, operating at an acceleration voltage of 200 kV. Images were recorded on the Gatan Rio 16 CMOS 4k camera (Gatan Inc., Pleasanton, CA, USA) and processed with Gatan Microscopy Suite (GMS) software (Gatan Inc., Pleasanton, CA, USA). Specimen preparation was carried out by vitrification of the aqueous solutions on grids with holey carbon film (Quantifoil R 2/2; Quantifoil Micro Tools GmbH, Großlöbichau, Germany). Before use, the grids were activated for 15 s in oxygen plasma using a Femto plasma cleaner (Diener Electronic, Ebhausen, Germany). Cryo-samples were prepared by applying a droplet (3 μL) of the suspension to the grid, blotting with filter paper and immediate freezing in liquid ethane using a fully automated blotting device Vitrobot Mark IV (Thermo Fisher Scientific, Waltham, MA, USA). After preparation, the vitrified specimens were kept under liquid nitrogen until they were inserted into a cryo-TEM-holder Gatan 626 (Gatan Inc., Pleasanton, CA, USA) and analyzed in the TEM at −178 °C.

### 2.6. In Vitro Drug Loading and Release Studies

Drug loaded micelles were prepared identically to blank micelles using 10 mg of copolymer, 1 mg of drug and 10 mL of DCM. Next, the unloaded drug was eliminated by filtration through a syringe filter (0.2 μm, Sartorius), the drug-loaded micelles solution was lyophilized, and then stored at −4 °C in the glovebox. To determine the drug loading efficiency (DLE) and drug loading content (DLC) the lyophilized drug-loaded micelles were redissolved in DMSO and analyzed with a UV-Vis-NIR spectrophotometer JASCO V-570 (Tokyo, Japan) using a standard curve method. The DLE and DLC were calculated according to the following equations:

$$\mathrm{Drug}\;\mathrm{loading}\;\mathrm{efficiency}\;\left(\mathrm{DLE}\%\right)=\frac{\mathrm{weight}\;\mathrm{of}\;\mathrm{drug}\;\mathrm{in}\;\mathrm{micelles}}{\mathrm{weight}\;\mathrm{of}\;\mathrm{drug}\;\mathrm{in}\;\mathrm{feed}}\times 100\%$$

$$\mathrm{Drug}\;\mathrm{loading}\;\mathrm{content}\;\left(\mathrm{DLC}\%\right)=\frac{\mathrm{weight}\;\mathrm{of}\;\mathrm{drug}\;\mathrm{in}\;\mathrm{micelles}}{\mathrm{weight}\;\mathrm{of}\;\mathrm{drug}\;\mathrm{loaded}\;\mathrm{micelles}\;}\times 100\%$$

In vitro drug release from micelles was carried out in PBS at pH 7.4, 6.4, and 5.5 at 37 °C via a dialysis method. Typically, 3 mL (1 mg · mL^−1^) of drug-loaded micelles were transferred into a Float-A-Lyzer G2 dialysis device (MWCO 7000). Each dialysis device was immersed into 150 mL of corresponding buffer at 37 °C, stirring at 100 rpm. At predetermined time intervals, 50 μL of the solution was withdrawn from the dialysis device and replaced with an equal amount of fresh buffer. Quantitative assessment of the released drugs was carried out by UV-Vis spectroscopy. The release experiments were conducted in triplicate, and the results presented are the average of the data.

### 2.7. MTT Assay

Cell viability was assessed by the MTT assay (Merck, Darmstadt, Germany). Stock solutions of used compounds (glycoconjugates or DOX) were prepared in DMSO (DMSO content in the highest concentration did not exceed 0.5%) and diluted to the desired concentrations with the culture medium directly before the experiment. Blank micelles and drug-loaded micelle solutions were also diluted to the desired concentrations with the culture medium directly before the experiment. The cells were seeded into 96-well plates at concentrations of 5 × 10^3^ (MCF-7, NHDF-Neo) or 2 × 10^3^ (HCT-116) per well and incubated for 24 h in a humidified atmosphere of 5% CO_2_ at 37 °C. Next, the culture medium was removed and replaced with a solution of the tested compounds in a medium with varying concentrations. Cells treated with DMSO in the fresh medium (0.5%) were used as controls. Then cells were incubated with compounds for a further 24 h, 48 h, or 72 h. After that, the solutions were removed and the MTT solution (50 μL, 0.5 mg · mL^−1^ in PBS) was added into each well. After 3 h of incubation, the MTT solution was removed and the precipitated formazan crystals were dissolved in DMSO. The absorbance was measured spectrophotometrically at a 570 nm wavelength using the plate reader (Epoch, BioTek, Winooski, VT, USA). The experiment was conducted in at least three independent repetitions with four technical repeats for each tested concentration. The results were expressed as the survival fraction (%) of the control. The IC_50_ values were calculated using CalcuSyn software (version 2.0, Biosoft, Cambridge, UK). The IC_50_ parameter was defined as the concentration of the drug that was necessary to reduce the proliferation of cells to 50% of the untreated control. The results are shown as the average value ± standard deviation (SD).

### 2.8. Apoptosis and Cell Cycle Analyses

The cell cycle and the type of cell death induced by the test compounds were measured by using flow cytometry. The cells were seeded into 6-well plates at concentrations of 3 × 10^5^ (HCT-116) or 4 × 10^5^ (MCF-7, NHDF-Neo) per well and incubated for 24 h in a humidified atmosphere of 5% CO_2_ at 37 °C. Then, the culture medium was removed and replaced with a solution of the tested compounds at a concentration equal to the IC_50_ value. Cells treated with blank micelle were also tested. The untreated cells with fresh medium were used as the control. After 24 h of incubation, the solutions were removed and the cells were collected by trypsinization. Then cells were centrifuged (3 min, 2000 rpm, RT) and washed with PBS. Then the cells were centrifuged again (3 min, 2000 rpm, RT) and the supernatant was removed. The resulting pellet contained cells, which were used for flow cytometric experiments. The fraction of dead cells after treatment with the test compounds were detected using the Annexin-V apoptosis assay (BioLegend, San Diego, CA, USA) and propidium iodide (PI) solution (100 μg/mL (Merck, Darmstadt, Germany)) uptake test. Cells were suspended in 50 μL of cold Annexin-V binding buffer and incubated in the dark with 2.5 μL of antibody Annexin-V FITC and 10 μL of PI solution (3 mg/mL) for 20 min at 37 °C. After this time, 250 μL of the Annexin-V binding buffer was added and the samples were incubated in the dark on ice for 15 min. Cytometric analyses were performed immediately using an Aria III flow cytometer (Becton Dickinson, Franklin Lakes, NJ, USA) with the FITC configuration (488 nm excitation; emission: LP mirror 503, BP filter 530/30) or PE configuration (547 nm excitation; emission: 585 nm) and at least 10,000 cells were counted. 

For cell cycle analysis, the cells were suspended in 250 μL of hypotonic buffer (consisting of: sodium citrate dihydrate 1 g/L, propidium iodide (PI) 1 mg/mL, RNAse A 10 mg/mL, Triton X-100 1:9) and incubated in the dark at rt for 20 min. Measurement was performed using an Aria III flow cytometer (Becton Dickinson, USA), using at least 10,000 cells per sample. The cytofluorimetric configurations used are described above. The obtained data were analyzed using Flowing Software (Cell Imaging Core, Turku Center for Biotechnology, Turku, Finland).

### 2.9. Cell Microscopy Imaging

Microscopic imaging of cells was performed using fluorescence microscopy (Olympus FV1000 microscope). The cells were seeded into 35 mm Glass Bottom Dishes (MatTek Corporation) at a concentration of 5 × 10^4^ (HCT-116, MCF-7, NHDF-Neo) in 2 mL of medium and incubated for 24 h in a humidified atmosphere of 5% CO_2_ at 37 °C. Then, the culture medium was removed and replaced with a solution of the free DOX or DOX-loaded micelles in a medium at a dose of 1 µM (concentration equal to the previously calculated IC_50_ value). The untreated cells with the fresh medium were used as controls. After 24 h of incubation, 2 µL of Hoechst 33342 dye was added to the medium and incubated in the dark for 20 min at 37 °C. Then, the solutions were removed and the cells were washed with PBS. To each dish, 500 μL of PBS was introduced, then the cells were covered with a coverslip and observed using a fluorescence microscope. All imaging conditions, including laser power, photomultiplier tube, and offset settings, were aligned with the fluorescence intensity of the sample. Images were analyzed using free software ImageJ 1.47v (Wayne Rasband, National Institute of Health, Bethesda, MD, USA).

### 2.10. Statistical Analysis

For biological evaluation, at least three replicates were performed for every kind of experiment. The results were presented as the mean value +/− SD. The statistical analysis was based on a *t*-test, and a *p*-value less than 0.05 was considered statistically significant, indicated on the figures with an asterisk.

## 3. Results and Discussion

### 3.1. Copolymer Synthesis and Characterization

**Figure 1:**
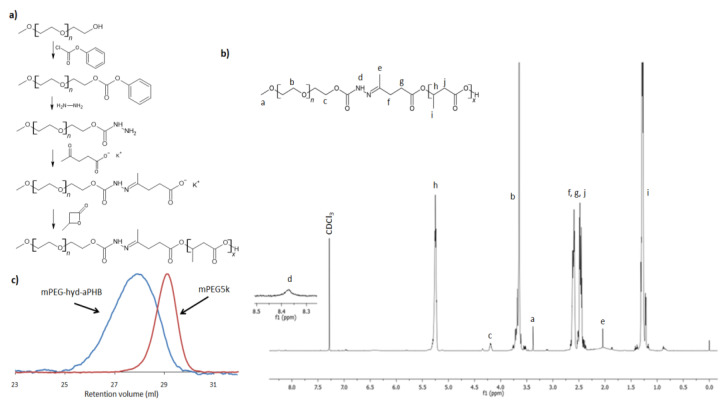
(**a**) The synthetic route for the mPEG-hyd-aPHB copolymer; (**b**) ^1^H NMR spectrum (CDCl_3_, 600 MHz) of mPEG-hyd-aPHB; (**c**) SEC traces of mPEG_5000_ and mPEG-hyd-aPHB.

The synthetic route of mPEG-hyd-aPHB is shown in Figure 1a. The essential issue in the design of shell-shedding micelles was the location of the pH-sensitive linkage between the hydrophilic and hydrophobic blocks. Herein, an acid-labile hydrazone bond was incorporated into the macroinitiator structure as described below. Namely, the amine-terminated monomethoxy poly(ethylene glycol) (M_n_ = 5000 g mol^−1^) (mPEG-NH-NH_2_) was first synthesized in two steps. Then, mPEG-NH-NH_2_ was reacted with levulinic acid potassium salt to obtain a hydrazone-containing macroinitiator. Subsequently, chain extension via anionic ring-opening polymerization of β-butyrolactone afforded the desired amphiphilic copolymer. 

Firstly, the hydroxyl end group of mPEG was activated through a reaction with *p*-nitrophenyl chloroformate to obtain mPEG-NPC. Upon activation of the mPEG hydroxyl end group with p-NPC, the ^1^H NMR spectrum (Figure S2a) revealed signals of the mPEG chain (3.65 and 3.38 ppm) and derived signals at δ = 7.4 and 8.28 ppm from aromatic protons of the *p*-nitrophenyl group, as well as a peak at 4.44 ppm attributed to substitution of mPEG with the NPC terminal methylene group. Then, the mPEG-NPC was reacted with hydrazine to obtain mPEG-NH-NH_2_. The disappearance of the aromatic *p*-NPC signals and the shifting of the signal of a methylene group from δ = 4.44 ppm to 4.27 ppm, observed in ^1^H NMR spectrum of amine-terminated mPEG (Figure S2b), confirms the mPEG-NH-NH_2_ formation. Subsequently, amino-terminated mPEG was further reacted with levulinic acid potassium salt. In a facile amine-ketone coupling reaction, a hydrazone bond containing potassium carboxylate macroninitiator was formed. The chemical structure of the macroinitiator (mPEG-hyd-LEV) was verified by ^1^H NMR, which is shown in Figure S2c. The presence of characteristic signals derived from incorporated levulinic acid potassium salt was observed. Importantly, the signal of the methyl group of potassium levulinic acid salt (see inset in Figure S2c) shifted from δ = 2.20 ppm to 1.99 ppm, indicating hydrazone bond formation. The same effect was observed for the signals ascribed to levulinic acid methylene groups (triplets at δ = 2.62 and 2.76 ppm) that overlapped after the formation of the hydrazone bond and were observed as a multiplet at δ = 2.47 ppm. Finally, the pH-responsive amphiphilic poly(ethylene glycol)-hyd-poly([R,S]-3-hydroxybutyrate) copolymer (mPEG-hyd-aPHB) was synthesized by anionic ring-opening polymerization of β-butyrolactone initiated with obtained hydrazone linkage-containing macroinitiator. In aqueous media, the amphiphilic block copolymers, depending on the copolymer hydrophilic block weight fraction (*f*), molar mass, and the interactions of the hydrophobic copolymer block with water may self-organize into micelles [20]. The hydrophilic block weight fraction usually affects the hydrophobic interactions and size of self-assembled structures and, if *f* is in the range of 45–75%, micelles are usually formed [47]. Therefore, the molar mass of poly([R,S]-3-hydroxybutyrate) block was planned to be 3500 g · mol^−1^ to keep the mPEG, i.e., hydrophilic block weight fraction, at the level of 60%. In β-butyrolactone anionic ring-opening polymerization, the initiation reaction by a weak nucleophile, e.g., carboxylic acid salt, the initiator attacks the C4 carbon of the β-butyrolactone ring by breaking the alkyl-oxygen bonds. As a result, the carboxylate ions formed in this reaction are the propagation centers. The activity of the initiator and the propagation species in such polymerization strongly depend on counter-ion size, cation-anion interactions, and solvent polarity [48]. Playing with the counterion size and solvent polarity, the activity of the initiating system and the reaction kinetics can be easily adjusted. For the sake of simplicity and efficiency, a reaction system containing a potassium counter-ion and DMSO as a solvent was chosen to obtain the appropriate polymerization conditions. The DMSO as a high-polar solvent activates the carboxylate species by strong solvation of the cation, resulting in significant increases in the reactivity of the system. As a result, the kinetics of the reaction allow for a controlled polymerization process on carboxylate growing species. The ^1^H NMR spectrum of mPEG-hyd-aPHB is shown in Figure 1b. All of the main characteristic signals assigned to mPEG (δ = 3.38 and 3.65 ppm) and methylene group of hydrazone-functionalized levulinic acid at δ = 2.02 ppm and aPHB at δ = 1.28, 2.54, 5.25 ppm) were observed. Using integral ratio with the molar mass of the mPEG block (5000 g mol^−1^), the molar mass of mPEG-hyd-aPHB was determined to be 8600 g mol^−1^. This value correlates with the theoretical one, 8600 g mol^−1^. SEC analysis, shown in Figure 1c, revealed that the obtained copolymer had a molar mass of 8200 g mol^−1^ with a dispersity value *Đ* = 1.14. It is worth mentioning that very low-intensity signals at δ = 1.86, 5.8 and 6.95 ppm in the ^1^H NMR spectrum of the copolymer were ascribed to the crotonate protons, which are a result of the abstraction of an acidic proton either from β-butyrolactone or aPHB (Figure S4) [13]. However, the amount is deemed to be negligible. The mPEG-*b*-aPHB diblock copolymer with a similar composition was also synthesized (Figure S3) and used as a pH-insensitive control in further studies. The results of the ^1^H NMR and SEC analyses for the synthesized copolymers are summarized in Table 1.

### 3.2. Self-Assembly of the Copolymers

**Figure 2:**
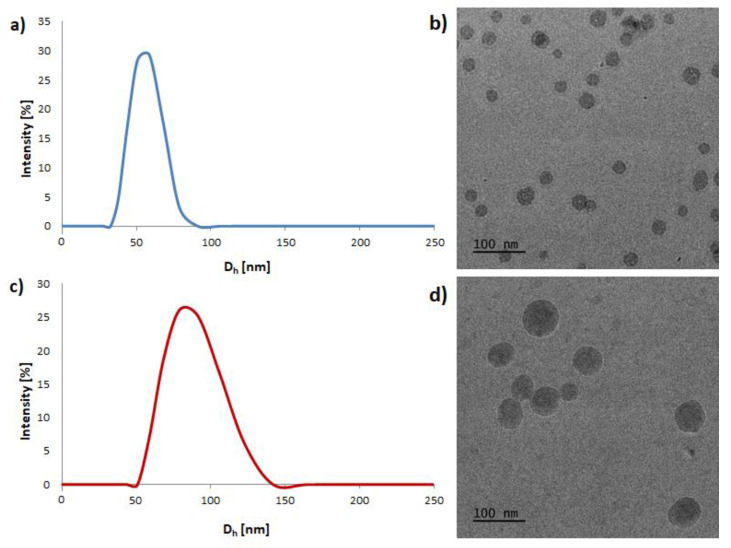
The size distribution and cryo-TEM images of mPEG-hyd-aPHB micelles (**a**,**b**) and 8HQ-loaded mPEG-hyd-aPHB micelles (**c**,**d**).

The synthesized amphiphilic copolymers spontaneously aggregated into core-shell structured micelles in an aqueous solution. Herein, the corresponding copolymers self-assembled into micelles, in which a core consists of hydrophobic aPHB blocks and an outer shell containing hydrophilic PEG chains. The structures of pH-responsive mPEG-hyd-aPHB and non-pH-responsive mPEG-*b*-aPHB micelles were confirmed using ^1^H NMR spectroscopy. Figure S5 shows the ^1^H NMR spectra corresponding to the lyophilized micelles that were resuspended in D_2_O. After micellization, the signals ascribed to aPHB blocks are barely visible, indicating that aPHB chains are located in the core of the micelles. Moreover, peaks corresponding to PEG protons are visible after self-organizing because poly(ethylene glycol) chains are in an outer mobile state. Similar results were observed for both mPEG-hyd-aPHB and mPEG-*b*-aPHB micelles. The critical micelle concentration was determined, using pyrene as a probe, from the plot of the excitation intensity ratio as a function of the copolymer concentration (Figure S6). The CMC values of mPEG-hyd-aPHB and mPEG-b-aPHB were determined to be 3.6 µg mL^−1^ and 2.2 µg mL^−1^, respectively (Table 1). The obtained micelles were characterized by DLS and cryo-TEM. As shown in Figure 2, the mPEG-hyd-aPHB formed micelles with a hydrodynamic diameter of ~55 nm and with a narrow size distribution (PDI = 0.14). In contrast, the hydrodynamic diameter for DOX-loaded mPEG-hyd-aPHB increased to ~86 nm, with a simultaneous increase in the PDI value up to 0.22. This increase in the hydrodynamic diameter of the micelles could be caused by drug encapsulation in the core. Additionally, it significantly increases the PDI values for drug-loaded micelles (Table 2). Next, cryo-TEM studies were used to observe the morphology of the micelles, revealing that the micelles had regular spherical shapes. The mean hydrodynamic diameters of nanoparticles determined by cryo-TEM were slightly lower compared with the values revealed by DLS, since the diameter obtained from DLS studies shows the hydrodynamic diameters in a hydrated state in the solution, while the diameter observed by cryo-TEM indicates the same for dried micelles [49]. 

### 3.3. Drug Loading and In Vitro Release Studies

**Figure 3:**
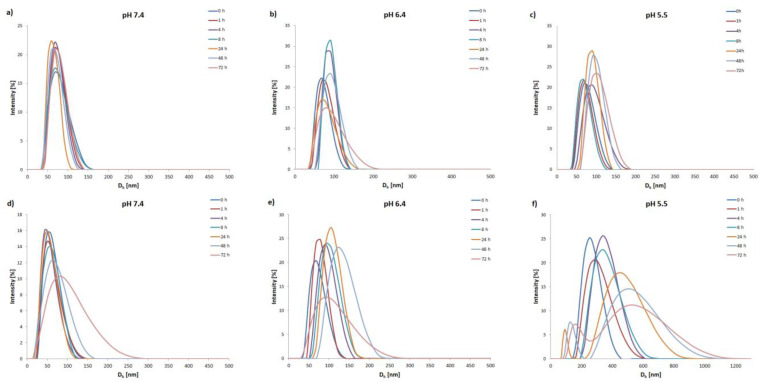
Changes in hydrodynamic diameter (d, nm) of mPEG-b-aPHB micelles (**a**–**c**) and mPEG-hyd-aPHB micelles (**d**–**f**) in PBS buffers at different pHs determined by DLS.

To examine the pH-triggered degradation of mPEG-hyd-aPHB micelles, the DLS technique was used to monitor the size changes in the micelles under various acidic conditions (PBS buffers with pH 7.4, 6.4, and 5.5) at 37 °C. In addition, non-pH-sensitive mPEG-*b*-aPHB micelles were also studied as a control. As shown in Figure 3, at physiological pH (7.4) the mPEG-hyd-aPHB micelles remained stable up to 24 h. After this, micelles slowly start forming larger aggregates, probably due to very slow hydrolysis of hydrazone linkage followed by reorganization. Under a mildly acidic environment (pH 6.4) mPEG-hyd-aPHB micelles started reorganizing into larger structures after 2 h, and larger micelles with hydrodynamic diameters of ~150 nm were formed over 48 h. Furthermore, as shown in Figure 3f, hydrazone bond-containing micelles immediately after dispersion in pH 5.5 buffer become unstable and form >200 nm particles (it took about 15 min to disperse the freeze-dried micelles in the corresponding buffer for measurement—which corresponds to sample “0 h” in the graph in all cases). Within 2 h, the hydrodynamic diameter increased up to >300 nm. After 24 h, populations from 100 to 800 nm were observed, meaning that the destruction of the particles had occurred. The observed phenomenon may be attributed to the hydrolysis of the hydrazone linkage, which leads to shedding of the hydrophilic PEG chains from the micelle and subsequent aggregation of hydrophobic core-forming chains into larger agglomerates [50]. In the case of control studies using non-pH-sensitive mPEG-*b*-aPHB micelles, no significant changes in the hydrodynamic diameters were observed. The obtained results indicate that mPEG-hyd-aPHB micelles are stable in the physiological environment and possess pH-responsive properties.

The drug-loading and in vitro release studies were carried out to evaluate the feasibility of using mPEG-hyd-aPHB nanocarriers as a pH-responsive anticancer drug delivery system. It is well-known that the hydrophobic core of micelles can encapsulate hydrophobic drugs. In this work, 8-hydroxyquinoline (8HQ), 8-hydroxyquinoline glucose conjugate (8HQ-Glu), 8-hydroxyquinoline galactose conjugate (8HQ-Gal), and doxorubicin (DOX) were used as hydrophobic model drugs to evaluate drug-loading properties and in vitro pH-dependent release behaviors from mPEG-hyd-aPHB and mPEG-*b*-aPHB micelles. The drug to copolymer ratio was 1:10. The drug loading efficiency (DLE) and drug loading content (DLC) are listed in Table 2. It shows that, in the case of loading doxorubicin or both glycoconjugates, the DLC and DLE values show no significant differences. The DLC and DLE values of DOX-loaded mPEG-hyd-aPHB micelles were 5.3% and 48.6%, while the values for 8HQ-Glu and 8HQ-Gal were 5.2%, 45.6%, and 5.3%, 46.4% respectively. Nevertheless, the DLC and DLE of 8HQ are slightly higher at 6% and 55.1% respectively. This discrepancy may be due to the fact that highly hydrophobic low-molecular weight compounds generally show better loading properties [51]. 

**Figure 4:**
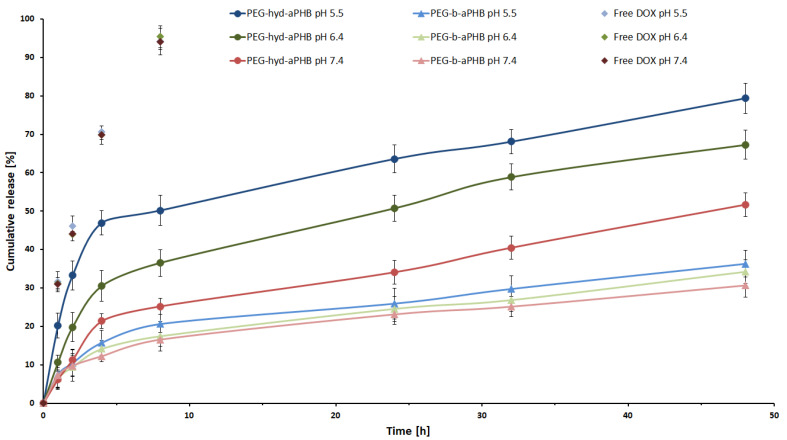
In vitro release profiles for DOX from mPEG-hyd-aPHB micelles, mPEG-b-aPHB micelles and free DOX in PBS buffers at 37 °C. Data are presented as the mean ± SD (*n* = 3).

The in vitro drug release profiles of mPEG-hyd-aPHB and mPEG-*b*-aPHB micelles were studied under three different buffer solutions of pH 7.4, 6.4, and 5.5, respectively, at 37 °C. Figure 4 shows the doxorubicin release profiles for the pH-responsive and control micelles at different pH values. These profiles showed that, at the physiological pH, encapsulated DOX was released from mPEG-hyd-aPHB micelles at levels of 21% and 40% after 8 h and 32 h, respectively. Under mildly acidic conditions (pH 6.4) corresponding to the extracellular pH of tumor tissues, the drug was released faster, i.e., 37% and 51% after 8 and 24 h, respectively. However, at pH 5.5 (the endo-lysosomal pH in cancer cells) the burst release of DOX was observed with 47% of the drug released after 8 h. The complete release of DOX from mPEG-hyd-aPHB micelles was observed after 48 h, whereas 79% of the drug was released. The control mPEG-*b*-aPHB micelles displayed a slower sustained release profile under all studied pH conditions. Less than ~36% of the DOX was released over 48 h in all pH values. The slight differences in the released DOX for mPEG-*b*-aPHB are most likely due to the hydrophobic DOX solubility increases with lowered pH [52]. For comparison, the effect of the matrix was also examined, the cumulative release of the free drug showed a release of 70% and 94%, within 4 and 8 h separately from pH conditions. The above results demonstrate that encapsulated drug release from mPEG-hyd-aPHB was significantly faster in acidic conditions as a result of accelerated hydrolysis of hydrazone linkage.

### 3.4. In Vitro Cytotoxicity Assay

It has been well-reported that tumor tissues have an increased demand for metal ions such as copper, iron, zinc, etc., since these microelements are involved in many cellular processes. Therefore, ion binding agents acting as ionophores are considered a promising therapeutic strategy in clinical practice [53,54]. Especially, the 8-hydroxyquinoline (8-HQ) scaffold is a privileged structure used in designing new active agents with therapeutic potential because of its ability of chelating metals [55]. On the other hand, the 8-HQ scaffold possesses poor solubility, bioavailability, and pharmacokinetic parameters [56]. The major challenge in the development of novel anticancer compounds is their selectivity towards tumor tissues. Therefore, it seems rational to use all abnormalities of cancer cells to increase the selectivity of anticancer therapies. Taking advantage of the Warburg effect, the 8-HQ derivatives have been successfully modified by conjugation with different sugar units to improve intramembrane transport and selectivity in drug targeting [57,58]. On the other hand, it has been widely described in the literature that tumor tissues are heterogeneous, and the levels of overexpression in GLUT transporters are different from cell to cell [59]. Therefore, the combination of glycoconjugation with pH-responsive nanocarriers to exploit the tumor-specific Warburg effect should improve the selectivity of anticancer therapy. 

In the present studies, the cytotoxic activity in vitro of mPEG-hyd-aPHB blank micelles as well as model drugs, i.e., 8-hydroxyquinoline (8HQ), glucose-conjugate 8-hydroxyquinoline (8HQ-Glu), galactose-conjugate 8-hydroxyquinoline (8HQ-Gal), doxorubicin (DOX), and drug-loaded micelles (DOX-micelles, 8HQ-mic, 8HQ-Glu-mic, and 8HQ-Gal-mic) was evaluated by MTT assay. The selectivity of compounds was tested on two cancer cell lines: HCT-116 (colorectal carcinoma cell line) and MCF-7 (human breast adenocarcinoma cell line), as well as, for comparison, on a healthy cell line—Normal Human Dermal Fibroblasts-Neonatal (NHDF-Neo). Overexpression of GLUT transporters as well as a slightly acidic microenvironment have been observed in these cell lines [60,61,62]. Cells were incubated with the appropriate free drugs or drug-loaded micelles for 24, 48, and 72 h in a concentration range oscillating within their IC_50_ activity.

**Figure 5:**
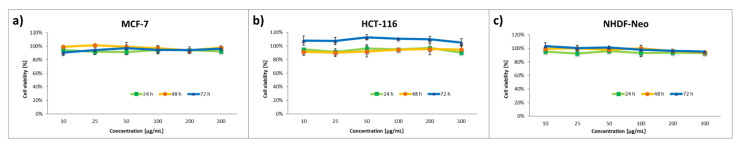
Cytotoxicity of the blank micelles on corresponding cells: (**a**) MCF-7, (**b**) HCT-116, (**c**) NHDF-Neo. Data are presented as the mean ± SD (*n* = 3).

In the beginning, the effect of blank micelles on cell viability was investigated (Figure 5). The results of the cytotoxicity assay indicate that the blank micelles in the concentration range tested were non-toxic to the tested cell lines, and therefore are not able to inhibit the proliferation of cancer as well as normal cells. This confirms that these nanocarriers could be safely used for research on the drug delivery system targeting cancer. The viability of NHDF cells during incubation with the blank micelles remained in the range of 92–103% at all of the tested concentrations, even after 72 h of the experiment.

**Figure 6:**
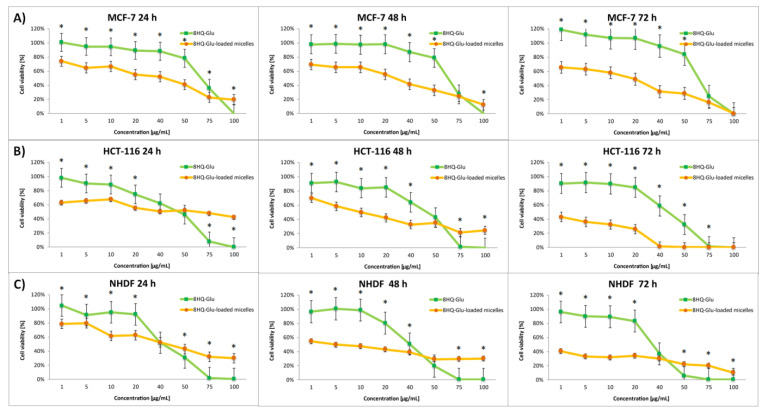
Cytotoxicity of different doses of free 8HQ-Glu and 8HQ-Glu-loaded micelles for MCF-7 (**A**), HCT-116 (**B**), and NHDF-Neo (**C**) cells after 24 h, 48 h, and 72 h of incubation. Data are presented as the mean ± SD (*n* = 3). The statistical significance between compounds was calculated with a *t*-test with *p*-value < 0.05, and indicated by an asterisk.

**Figure 7:**
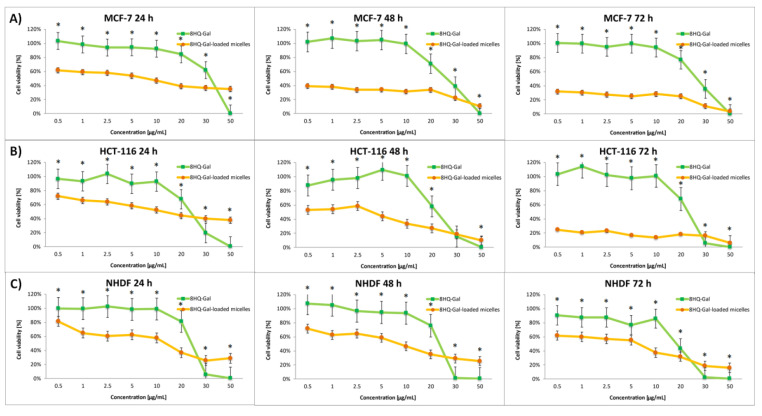
Cytotoxicity of different doses of free 8HQ-Gal and 8HQ-Gal-loaded micelles for MCF-7 (**A**), HCT-116 (**B**), and NHDF-Neo (**C**) cells after 24 h, 48 h, and 72 h of incubation. Data are presented as the mean ± SD (*n* = 3). The statistical significance between compounds was calculated with a *t*-test with *p*-value < 0.05, and indicated by an asterisk.

**Figure 8:**
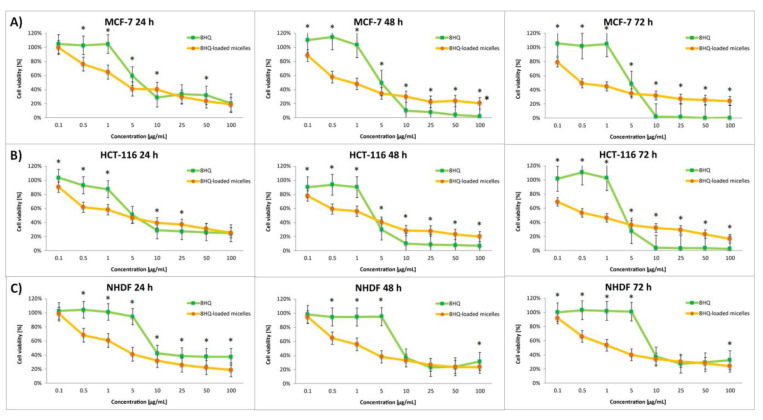
Cytotoxicity of different doses of free 8HQ and 8HQ-loaded micelles for MCF-7 (**A**), HCT-116 (**B**), and NHDF-Neo (**C**) cells after 24 h, 48 h, and 72 h of incubation. Data are presented as the mean ± SD (*n* = 3). The statistical significance between compounds was calculated with a *t*-test with *p*-value < 0.05, and indicated by an asterisk.

**Figure 9:**
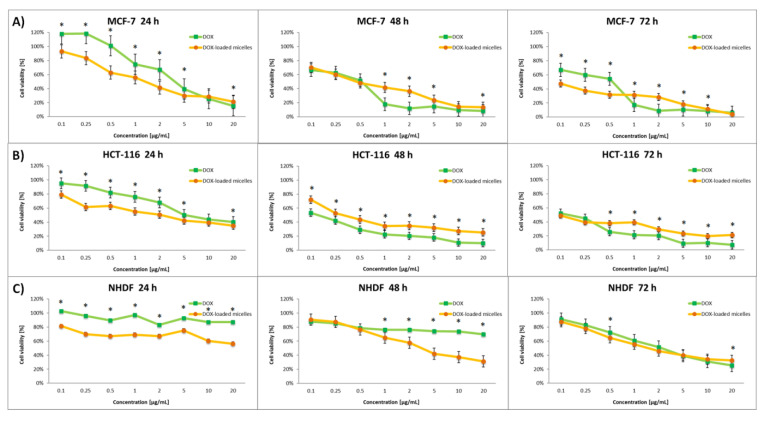
Cytotoxicity of different doses of free DOX and DOX-loaded micelles for MCF-7 (**A**), HCT-116 (**B**), and NHDF-Neo (**C**) cells after 24 h, 48 h, and 72 h of incubation. Data are presented as the mean ± SD (*n* = 3). The statistical significance between compounds was calculated with a *t*-test with *p*-value < 0.05, and indicated by an asterisk.

The effect of the nanocarriers was assessed by comparing the cytotoxic activity of free drugs and drug-loaded micelles. The dose-dependent cytotoxic effect of each of the tested compounds is shown in Figure 6, Figure 7, Figure 8 and Figure 9. Results of the screening tests were used to determine the IC_50_ value, defined as 50% cell growth inhibition in comparison to the untreated control, and are summarized in Table 3. The results showed that free 8HQ-Glu and 8HQ-Gal exhibit constant activity over time against all cell lines tested. The IC_50_ values obtained for free glycoconjugates are mostly higher than for their micelle form (Table 3). The lower cytotoxic activity of free glycoconjugates is probably caused by their hindered penetration through the cell membranes. In the case of drug-loaded micelles, the cell viability decreases with incubation time, which is not noticeable when free glycoconjugates are incubated. This is due to the gradual slow release of the drug from the micelles. The cytotoxicity of the glycoconjugate will increase with the degree of release of the drug from the polymeric carriers. The closure of glycoconjugates in micelles significantly improved their cytotoxic activity—the compound exhibited a strong effect at low doses.

The main challenge in the development of new pharmaceuticals is their selectivity. The optimal drug should target only cancer cells and save healthy ones. Unfortunately, the tested glycoconjugates (8HQ-Glu and 8HQ-Gal) turned out to be toxic also to healthy cells (NHDF-Neo). The lack of selectivity is probably due to the mechanism by which the drug penetrates the cell. Table 4 presents the selectivity index of the tested compounds after 72 h of incubation. The selectivity index (SI) was calculated as a ratio of the IC_50_ value determined for normal cells to the IC_50_ value determined for cancer cells. The higher the SI, the safer and more selective the test compound is. It is worth noting that 8HQ-Glu-mic and 8HQ-Gal-mic showed high selectivity towards HCT-116 cells (SI = 35.21 and 164.12, respectively) and slightly lower selectivity towards MCF-7 cells (SI = 22.68 and 14.68, respectively).

The above results suggest a different kind of drug-loaded micelle interactions with normal cells compared to colon and breast cancer cells. The difference may be due to the environment surrounding the cells. The use of micelles allows the release of the drug in a low-pH environment, thereby increasing drug accumulation in the tumor and preventing early drug release into systemic circulation. Thus, hydrazone linkage could maintain stability in normal cells, but by taking advantage of the acidic environment near the tumor cells, the micelles can release the drug already near the tumor cell. On the other hand, the non-polar nature of the released glycoconjugates should facilitate the process of crossing the phospholipid bilayer through passive diffusion and cell penetration, where intracellular hydrolytic enzymes are then able to remove acetyl groups in sugar units. Due to the nature of the micelles, it is necessary to load them with hydrophobic compounds such as glycoconjugates with acetyl protection of the hydroxyl groups. One of the future possibilities to improve the selectivity even more is to develop a material that allows the closure of hydrophilic glycoconjugates with free OH groups in the sugar unit. Such compounds have the potential to penetrate the tumor cell by active transport through GLUT transporters. Another possibility is for micelles to enter the neoplastic cell by endocytosis. Then, there will be a gradual degradation of pH-sensitive micelles already in the tumor cell and the gradual release of cytotoxic drugs. In healthy tissue, the drug is released less or not at all, due to the less favorable environment not leading to the degradation of the nanocarriers.

To approximate the mechanism of compound penetration cells, experiments were performed with 8HQ. This compound shows poor bioavailability, so when released from micelles near the cancer cell, it should show a similar effect to free 8HQ due to the impeded ability of the compound to cross into the cell. The experiments showed that 8HQ-loaded micelles exhibited higher proliferation inhibition compared to free 8HQ toward HCT-116 and MCF-7 cells in the concentration range tested. In addition, the maximum cytotoxic dose of free 8HQ can be observed already after 48 h. In contrast, in the case of 8HQ-mic, the drug is released over time and causes the gradual death of tumor cells for 72 h of the experiment. Therefore, we are probably dealing with the penetration of micelles by endocytosis into the tumor cell and the slow release of the loaded 8HQ inside the cell. However, no mechanism of action can be excluded at this step. Additionally, more detailed studies are needed to determine the penetration mechanism and treatment options. It should be noted that, in the case of 8HQ and 8HQ-mic, there was only a slight improvement in the selectivity index. This suggests that both the micelles and the sugar fragment are needed to improve the activity of free 8HQ. Most of all, the addition of a sugar unit allowed improvement of the selectivity of the tested compounds (as can be noticed from the examples of the compounds 8HQ-Glu-mic and 8HQ-Gal-mic). Thus, the synergistic action of pH-responsive nanocarriers and glycoconjugation could exhibit better efficacy through higher toxicity and lower side effects.

Experiments have also been carried out on the standard drug used as an anticancer agent—doxorubicin. As with glycoconjugates, it has been found that the DOX is released gradually from the micelles. Finally, after 72 h of the experiment in vitro, DOX-loaded micelles showed comparable antiproliferative activity compared to unmodified free DOX against various cancer cell lines and a higher selectivity index in comparison to free DOX. The entrapment of the drug in the carriers enhances the therapeutic effect by protecting the drug from early degradation and prolongs the release of the reduced dose of the drug locally, not systemically.

### 3.5. Apoptosis and Cell Cycle Analyses by Flow Cytometry

**Figure 10:**
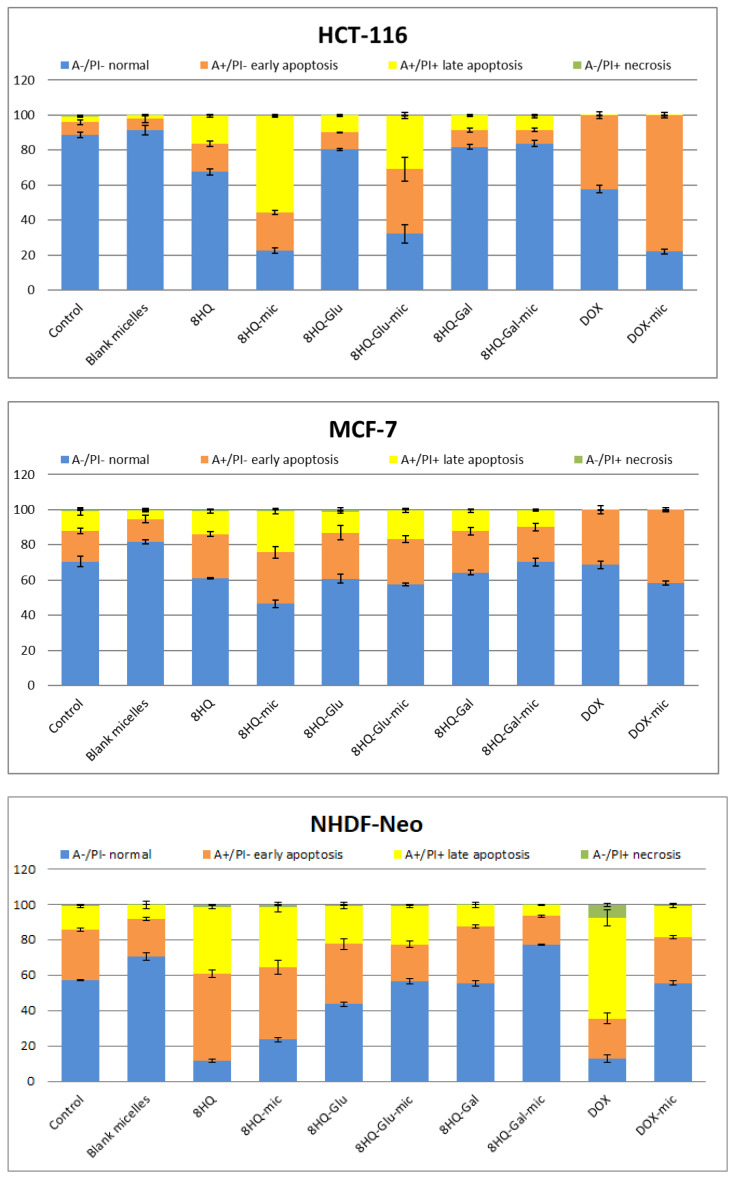
Flow cytometry analysis of HCT-116, MCF-7, and NHDF-Neo cell apoptosis induced by tested compounds at a dose equal to the previously calculated IC_50_ value after incubation for 24 h. Apoptotic effects as determined by Annexin V-FITC and PI assays. Data are presented as mean ± SD (*n* = 3).

After completing the MTT cytotoxicity test and determining the IC_50_ parameters for compounds, a test using Annexin V-FITC and Propidium Iodide (PI) was performed, aimed at determining what type of cell death is caused by the tested compounds. The compounds were selected at concentrations equal to their previously calculated IC_50_ value. The experiments were carried out for the MCF-7 and HCT-116 tumor cell line and the NHDF-Neo healthy cell line, for the one-time test of 24 h cell incubation with compound solutions. The results of the apoptosis analysis using the flow cytometry method are shown in Figure 10 and Figure S7. Apoptosis is a natural biological process involving programmed or suicidal cell death, which plays an important role in maintaining the correct homeostasis between proliferation and cell death. The test carried out allows the distinguishing of live cells from apoptotic and necrotic cell subpopulations, which is a source of important information on the cytotoxicity of chemicals. Annexin V-FITC labels the phosphatidylserine sites that, after the start of apoptosis, are located on an outer layer of the cell membrane, enabling the detection of early apoptosis. PI labels intracellular DNA (PI binds stoichiometrically to DNA) in cells during late apoptosis and necrosis, where the cell membrane has been disturbed. This combination allows for the differentiation of early apoptotic (A+/P−), late apoptotic (A+/P+), necrotic (A−/P+), and viable cells (A−/P−), which can be quantified by flow cytometry.

Figure 10 shows the level of induction of apoptosis by the tested compound. After 24 h of incubation, no necrotic conditions were detected in any of the cell lines used. On the other hand, the percentage of cells in both early and late apoptosis was observed. This means that the applied IC_50_ dose affects the entry of cells into the apoptosis pathway and does not cause uncontrolled pathological cell death as a result of mechanical damage. As can be seen, blank micelles, comparable to the untreated control group, show no apoptosis characteristics after 24 h of incubation. Thus, the results of the apoptosis test confirmed the negligible toxic effect of the nanocarriers on the cells tested. On the other hand, entrapment of drugs in carriers, in almost every case, increased the rate of apoptosis of tumor cells compared to free drugs. The highest rate of apoptosis in both cancer cell lines was recorded for 8HQ-mic (79% for HCT-116 and 52% for MCF-7, respectively), which was much higher than for free 8HQ (33% for HCT-116 and 40% for MCF-7). At the same time, this compound showed high toxicity also to healthy cells (76% for NHDF-Neo). These results are in agreement with the MTT cytotoxicity assays described previously. A high share of dead cells was also observed for the compounds 8HQ-Glu-mic (69% for HCT-116 and 42% for MCF-7) and DOX-mic (78% for HCT-116 and 42% for MCF-7). Moreover, 8HQ-Glu-mic and DOX-mic induce both early and late apoptosis much more effectively than free 8HQ-Glu (20% for HCT-116 and 41% for MCF-7) and free DOX (45% for HCT-116 and 34% for MCF-7). The opposite is true for the NHDF-Neo healthy cell line, where the entrapment of drugs in micelles resulted in lower toxicity to these cells. The percentage of apoptotic cells is higher after treating healthy cells with free drugs than with drug-loaded micelles. The results of the measurements performed do not show clear apoptotic changes for 8HQ-Gal and 8HQ-Gal-mic, which may be due to the short incubation time. Extending the incubation time to 72 h would probably give better results, which would be reflected in the results of the MTT cytotoxicity tests. A higher percentage of apoptotic cells was observed in the HCT-116 cell line, which is also consistent with the results of the MTT cytotoxicity assays. This suggests that the cells of the colon cancer line are particularly sensitive to the compounds tested. The insensitivity of MCF-7 cells can be explained by the short incubation time of cells with test drugs.

**Figure 11:**
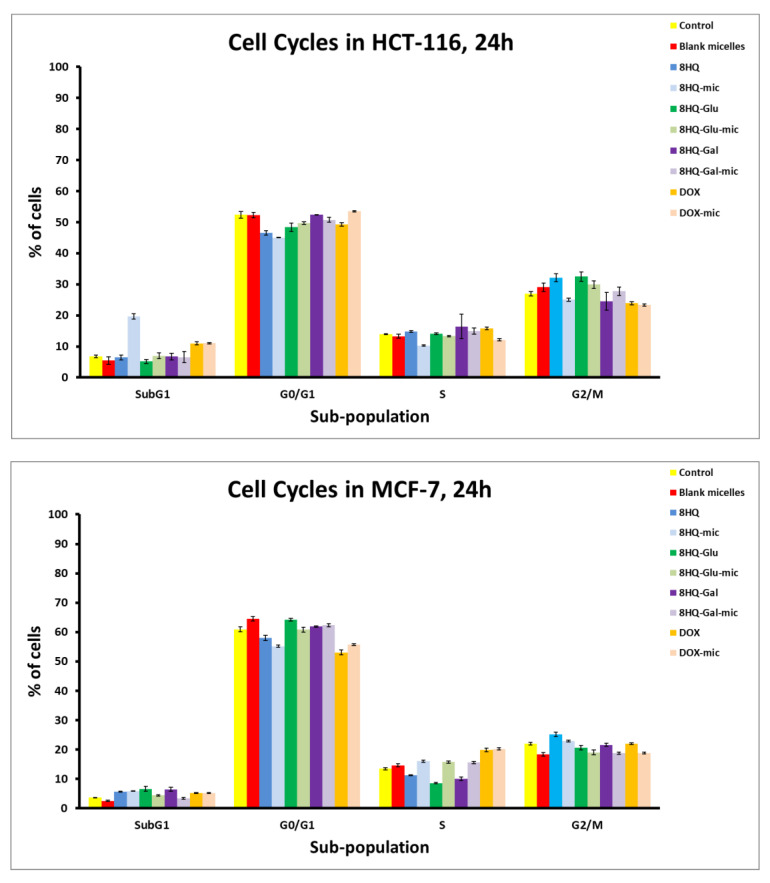

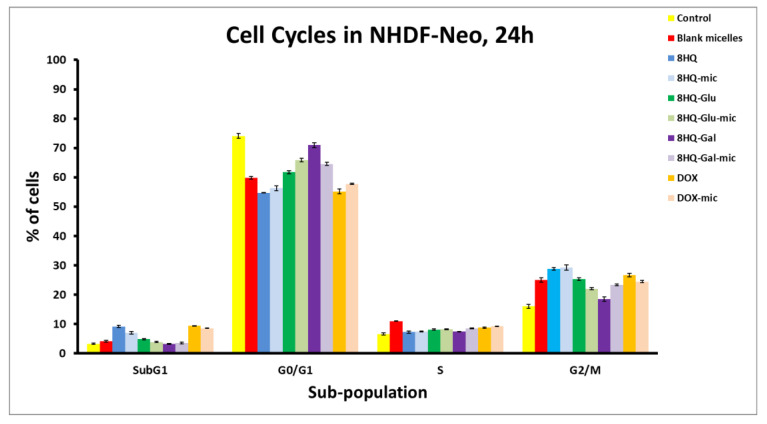
Flow cytometry analysis of HCT-116, MCF-7, and NHDF-NEO cell cycle distribution induced by tested drugs at a dose equal to the previously calculated IC_50_ value after incubation of 24 h. Data are presented as mean ± SD (*n* = 3).

After analyzing the type of cell death that is induced by the tested compounds, their influence on the progression of the cell cycle was investigated using the flow cytometry technique. The method of cell lysis and isolation of cell nuclei was used for the study. Namely, the collected cells were treated with a hypotonic buffer with a lysis effect, containing propidium iodide, which can bind to DNA by intercalation. It is known that cancer cells are characterized by uncontrolled cell proliferation. The cell cycle consists of the sequence of cell growth and division. The results of the analysis of the cell cycle distribution induced by tested drugs at a dose equal to the previously calculated IC_50_ value after incubation of 24 h are shown in Figure 11. Untreated cells were considered as controls. For the HCT-116 cell line, an increased Sub-G1 cellular fraction was observed after 24 h of incubation with the IC_50_ doses of 8HQ-mic as well as DOX and DOX-mic, which may suggest an increased sensitivity of the cells of this line to the tested prodrugs and the cytostatic potential of active substances released from the carriers. Cells in the sub-G1 phase correspond to apoptotic cells, so the results are in agreement with the results of the cell apoptosis analysis. Interestingly, the percentage of cells arrested in the Sub-G1 phase after treatment with 8HQ-loaded micelles is higher than for free 8HQ. The remaining compounds tested did not significantly affect the change of cell cycle phases as compared to the control. Cytometric evaluation for the MCF-7 cell line indicated that the effect of the tested compounds after 24 h of incubation did not significantly change the cell cycle phase distribution in comparison to the untreated control populations. The results are in agreement with the apoptosis and cytotoxicity studies performed previously. Perhaps this is caused by insufficient incubation time of the cells with the tested compounds. The results of tests performed on the healthy NHDF-Neo cell line showed cell cycle arrest in the Sub-G1 phase for the compounds 8HQ and 8HQ-mic, as well as DOX and DOX-mic. At the same time, the analyzes confirmed that neither the glycoconjugates nor the drug-loaded micelles caused any detectable cytotoxic effects. Taking into account the fact that the drugs at the IC_50_ dose were used in the research, inhibition of the NHDF-Neo cell cycle in the G2/M phase, which controls mitosis, was noticed. In general, cells are able to repair damage caused by drug doses. This reflects an increase in the G2/M phase, which does not result in the cell cycle arrest and inhibition of cell division, but a delay in the progression of the cell cycle due to mechanism repair processes that block the entry of cells into mitosis. It is worth emphasizing that the distribution of the cell cycle for the used carriers did not differ from the control data, which confirms the safety of their use in drug delivery systems. These experiments have shown that the developed micelles are safe, which is consistent with the earlier results of the MTT and Annexin V assays.

The results of the apoptosis and cell cycle analyses by flow cytometry confirmed the anticancer activity of the tested compounds and showed that they exhibit pro-apoptotic properties, without pro-inflammatory processes, and without affecting changes in the cell cycle distribution. The above results confirm that the use of nanocarriers increased the effectiveness of the compounds in the selective destruction of tumor cells, which can be attributed to the increased cellular uptake of carriers and the intracellular controlled release of the active molecules triggered by pH changes.

### 3.6. Cellular Uptake and Intracellular Drug Release

**Figure 12:**
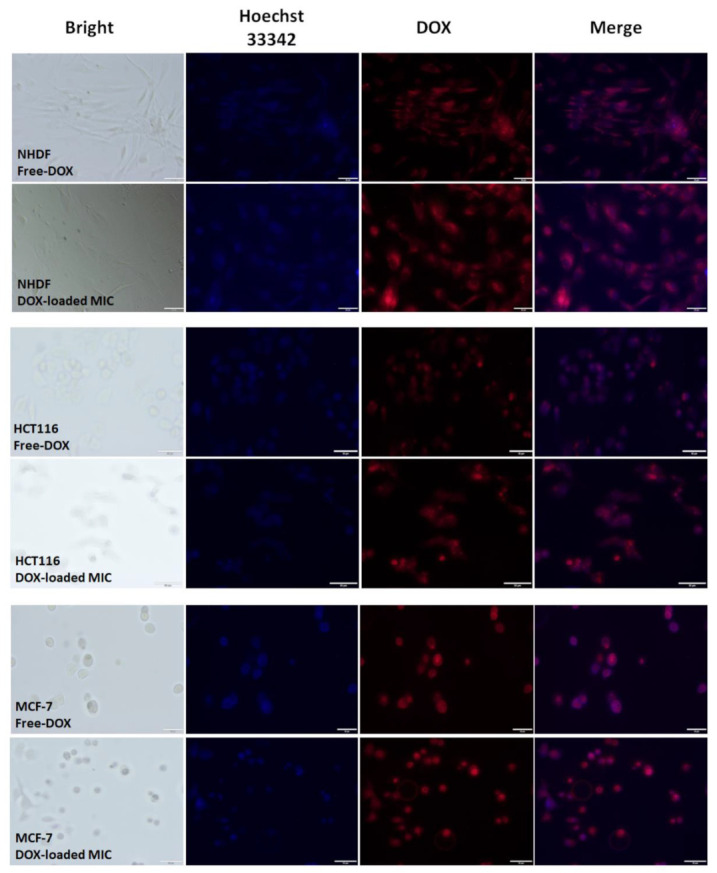
Microscopic images of MCF-7, HCT-116, and NHDF-Neo cells after 24 h of incubation with DOX and DOX-loaded mPEG-hyd-aPHB micelles (DOX dose: 1 μM). Images from left to right show cell nuclei stained by Hoechst 33342 (blue), DOX fluorescence in cells (red), and overlays of blue and red images. Scale bar: 50 μm.

Whether nanoparticles enter cells is an important factor in cancer therapy. The intracellular localization of DOX-loaded mPEG-hyd-aPHB micelles was investigated in both healthy cells (NHDF-Neo) and cancer cells (HCT116 and MCF-7) using fluorescence microscopy. Cells were incubated with DOX or DOX-loaded micelles for 24 h at a concentration of 1 µM (concentration equal to the previously calculated IC_50_ value). After incubation, the morphological characteristics of the cell nuclei were assessed by staining the cells with Hoechst 33342 dye, which passes through intact cell membranes and intercalates into DNA, then emits blue fluorescence. On the other hand, DOX, as a fluorescent compound, is visible in the red fluorescence channel. As shown in Figure 12, the free doxorubicin was found in the nuclei of all cell lines studied, as evidenced by a co-localization with the blue fluorescence of Hoechst 33342. However, DOX-loaded micelles were observed in the cytoplasm and nuclei. This can be attributed to the different cellular uptake mechanisms of free drugs and drug-loaded micelles. Free DOX was brought immediately into the cells through a passive diffusion mechanism, while nanosized DOX-loaded micelles were mostly internalized via an endocytosis process. Controlled release of the drug from the micelles, under the influence of pH, then delayed the delivery of DOX to the nucleus. However, the tumor’s acidic microenvironment could cause the breaking of the hydrazone linkage which could release the drug extracellularly. Because DOX itself is a fluorescent molecule, the intensity of red fluorescence in the cells should be equal to the amount of DOX uptake by cancer cells. The tested cancer cells (MCF-7 and HCT-116) treated with DOX-loaded mPEG-hyd-aPHB micelles revealed a higher intensity of red fluorescence in the nuclei compared to healthy NHDF-Neo cells, in which most of the DOX was observed in the cytoplasm. The observation could be attributed to the exhibition of intracellular drug-release behavior by the pH-responsive mPEG-hyd-aPHB micelles after they were internalized into cells. This is probably due to the faster release of DOX from micelles at acidic endo-lysosomal pH in cancer cells and the self-quenching effect of doxorubicin encapsulated in nanocarriers before release [63]. The acidic endo-lysosomal pH allows the cleavage of hydrazone bonds in mPEG-hyd-aPHB micelles, thus leading to prompt intracellular drug release from nanocarriers. The performed microscopic imaging confirmed that mPEG-hyd-aPHB nanocarriers could efficiently deliver DOX to tumor cells. The fluorescence intensity quantization method from the row images is presented in Supplementary File Figure S8. The signal intensity was measured, then followed by the digital quantization procedure presented previously [64].

## 4. Conclusions

In summary, biodegradable poly(ethylene glycol)-hyd-poly([R,S]-3-hydroxybutyrate) was synthesized via the anionic ring-opening polymerization of β-butyrolactone initiated by a hydrazone-functionalized PEG macroinitiator. The amphiphilic diblock copolymer could readily self-organize to form core-shell structured micelles (~70 nm determined by DLS and TEM) in an aqueous medium. The micelles were stable in physiological conditions (pH 7.4), while acid-triggered destruction of the hydrazone linkage caused shedding of the PEG chains, resulting in the release of the drug. In vitro drug release studies revealed that after 8 h, 21%, 37%, and 47% of the drug were released under pH 7.4, 6.4, and 5.5, respectively, demonstrating pH-dependent drug release behavior. The anticancer agents 8HQ, 8HQ-Glu, 8HQ-Gal, and DOX were effectively loaded into micelles to improve the metabolic stability and selectivity of tumor therapy. Red fluorescence signals, from the row images obtained after 24 h of incubation with micelles loaded with doxorubicin, were higher. The computational image preprocessing, as a quantization method, confirms better bioavailability of the novel anticancer agents. The in vitro cytotoxicity studies confirmed that blank mPEG-hyd-aPHB micelles are non-toxic towards HCT-116 and MCF-7 cells as well as, most importantly, towards healthy cell lines NHDF-Neo. Moreover, MTT assay results revealed that drug-loaded micelles efficiently inhibit cancer cell proliferation and promote the apoptosis of tumor cells. In some cases, especially after 72 h of incubation, 8HQ-glycoconjugates, which are still being investigated as potential anti-neoplastic agents, exhibit cytotoxicity comparable to the known drug DOX. However, even more importantly, the loading of drugs into micelles significantly increases their selectivity. This is particularly evident in the case of 8-HQ-glycoconjugates, for which the selectivity index in comparison to free drugs has increased, at best by up to two hundred times, and in other cases in the range of 19 to 48 times. The enhanced selectivity and antitumor efficacy of 8HQ-glycoconjugate-loaded micelles over free 8HQ are attributed to the joint effects of several factors, including taking advantage of the Warburg effect, i.e., the pH-triggered drug release, facilitated intermembrane transport of glycoconjugates, and prolonged circulation time or enhanced cellular internalization of nanocarriers by tumor cells. The strategy of a combination of pH-sensitive nanocarriers with glycoconjugation of the drug molecule provides an alternative for the design of sophisticated multi-stimuli nanocarriers to increase the selectivity of anticancer therapy. These promising in vitro results encourage the expansion of the panel of micelle loaded compounds, in which a sugar moiety has been attached to the drug in order to improve its selectivity. If subsequent results confirm the observations made so far, it will be advisable to select the best candidates for the next stage of in vivo tests.

## Figures and Tables

**Table 1:** Characteristics of copolymers.

Block Copolymer	Mn,_NMR_^a^ [g mol^−1^]	Mn,_SEC_^b^ [g mol^−1^]	*Đ*	*f*_PEG_ [%]	CMC ^c^ [µg mL^−1^]
mPEG-hyd-aPHB	8600	8200	1.14	58	3.6
mPEG-*b*-aPHB	8100	7900	1.08	62	2.2

^a^ The mPEG block had M_n_ = 5000 and *Đ* = 1.01 (determined by SEC). ^b^ Determined by SEC with DMF as solvent and poly(ethylene glycol) standards. ^c^ Determined using pyrene as a fluorescence probe.

**Table 2:** Characterization of blank and drug-loaded micelles.

Sample	mPEG-hyd-aPHB				mPEG-*b*-aPHB			
	Size [nm]	PDI	DLC [%]	DLE [%]	Size [nm]	PDI	DLC [%]	DLE [%]
Blank micelles	55.3 ± 2.8	0.14 ± 0.01	_	_	49.5 ± 4.1	0.18 ± 0.02	_	_
DOX-loaded micelles	85.7 ± 5.1	0.22 ± 0.03	5.3 ± 0.4	48.6 ± 3.1	82.8 ± 6.2	0.2 ± 0.01	5.7 ± 0.2	54.1 ± 3.8
8HQ-loaded micelles	77.7 ± 3.8	0.19 ± 0.02	6.0 ± 0.1	55.1 ± 1.9	87.9 ± 2.9	0.15 ± 0.02	5.8 ± 0.4	55.3 ± 2.3
8HQ-Glu-loaded micelles	104.9 ± 4.5	0.25 ± 0.03	5.2 ± 0.2	45.6 ± 2.2	100.8 ± 3.1	0.28 ± 0.04	5.1 ± 0.3	47.1 ± 2.6
8HQ-Gal-loaded micelles	92.5 ± 4.2	0.22 ± 0.02	5.3 ± 0.3	46.4 ± 2.5	99.9 ± 5.4	0.19 ± 0.02	5.3 ± 0.3	48.1 ± 3.2

**Table 3:** Screening of cytotoxicity of tested compounds against MCF-7, HCT-116, and NHDF-Neo cells for 24, 48, and 72 h incubation time.

**Compound**	**Activity IC_50_ [µM] ^a^**		
	**MCF-7**		
	**24 h**	**48 h**	**72 h**
DOX	3.42 ± 0.03	0.58 ± 0.02	0.57 ± 0.02
DOX-micelles	1.96 ± 0.01	0.52 ± 0.02	0.13 ± 0.01
8HQ	7.17 ± 0.21	2.86 ± 0.09	3.01 ± 0.05
8HQ-mic ^b^	4.82 ± 0.17	2.11 ± 0.11	0.75 ± 0.04
8HQ-Glu	65.86 ± 2.22	62.07 ± 1.84	63.57 ± 2.01
8HQ-Glu-mic ^c^	37.57 ± 0.92	3.85 ± 0.19	0.59 ± 0.02
8HQ-Gal	26.39 ± 1.28	23.15 ± 0.99	24.60 ± 0.52
8HQ-Gal-mic ^d^	5.59 ± 0.14	0.21 ± 0.01	0.19 ± 0.01
**Compound**	**Activity IC_50_ [µM] ^a^**		
	**HCT-116**		
	**24 h**	**48 h**	**72 h**
DOX	6.94 ± 0.33	0.095 ± 0.01	0.105 ± 0.01
DOX-micelles	2.36 ± 0.08	0.45 ± 0.01	0.078 ± 0.01
8HQ	9.33 ± 0.22	4.12 ± 0.12	4.40 ± 0.08
8HQ-mic ^b^	4.95 ± 0.16	1.80 ± 0.05	0.91 ± 0.04
8HQ-Glu	49.67 ± 1.32	47.20 ± 1.14	45.73 ± 1.81
8HQ-Glu-mic ^c^	49.60 ± 2.11	8.46 ± 0.12	0.38 ± 0.02
8HQ-Gal	22.10 ± 0.84	23.09 ± 0.92	22.52 ± 1.02
8HQ-Gal-mic ^d^	10.83 ± 0.33	2.64 ± 0.12	0.017 ± 0.01
**Compound**	**Activity IC_50_ [µM] ^a^**		
	**NHDF-Neo**		
	**24 h**	**48 h**	**72 h**
DOX	>20	>20	2.71 ± 0.08
DOX-micelles	>20	3.93 ± 0.12	2.50 ± 0.06
8HQ	9.34 ± 0.25	8.97 ± 0.19	9.64 ± 0.32
8HQ-mic ^b^	5.18 ± 0.26	3.96 ± 0.12	3.97 ± 0.09
8HQ-Glu	41.00 ± 2.01	41.11 ± 1.73	30.02 ± 0.94
8HQ-Glu-mic ^c^	41.70 ± 1.42	22.53 ± 0.65	13.38 ± 0.39
8HQ-Gal	20.14 ± 0.75	21.77 ± 0.89	18.30 ± 0.74
8HQ-Gal-mic ^d^	7.47 ± 0.17	5.55 ± 0.17	2.79 ± 0.13

^a^ Cytotoxicity was evaluated using the MTT assay; ^b^ 8HQ-loaded micelles; ^c^ 8HQ-Glu-loaded micelles; ^d^ 8HQ-Gal-loaded micelles. Data are presented as the mean ± SD (*n* = 3).

**Table 4:** Selectivity index (SI) of tested compounds after 72 h incubation time.

Compound	Selectivity Index (SI) ^a^	
	MCF-7	HCT-116
DOX	4.75	25.81
DOX-micelles	19.23	32.05
8HQ	3.20	2.19
8HQ-mic ^b^	5.29	4.36
8HQ-Glu	0.47	1.39
8HQ-Glu-mic ^c^	22.68	35.21
8HQ-Gal	0.74	0.81
8HQ-Gal-mic ^d^	14.68	164.12

^a^ Selectivity index (SI) was calculated as a ratio of the IC50 value for healthy cells (NHDF-Neo) and the IC50 value for cancer cells (MCF-7 or HCT-116); ^b^ 8HQ-loaded micelles; ^c^ 8HQ-Glu-loaded micelles; ^d^ 8HQ-Gal-loaded micelles.

## Data Availability

The raw data supporting the conclusions of this article will be made available upon request.

## Supplementary Materials

The following supporting information can be downloaded at: https://www.mdpi.com/article/10.3390/pharmaceutics14020290/s1, Figure S1: Structure of glycoconjugates (A) 8-((1-(2,3,4,6-tetra-O-acetyl-β-D-glucopyranosyl)-1H-1,2,3-triazol-4-yl)methoxy)quinoline (8HQ-glucose conjugate–8HQ-Glu) and (B) 8-((1-(2,3,4,6-tetra-O-acetyl-β-D-galactopyranosyl)-1H-1,2,3-triazol-4-yl)methoxy)quinoline (8HQ-galactose conjugate–8HQ-Gal); Figure S2: 1H NMR spectrum (CDCl3, 600 MHz) of PEG-NPC, PEG-NH-NH2 and PEG-hyd-LEV; Figure S3: (a) 1H NMR spectrum (CDCl3, 600 MHz) and (b) SEC traces of mPEG-b-aPHB; Figure S4: The possible routes of crotonate end-groups forming i.e. (i) abstraction of the acidic proton of a monomer methylene group with formation of lactone enolate, followed by elimination reaction to form a crotonate anion, which initiates further polymerization anionic ring-opening polymerization of β-butyrolactone; (ii) chain transfer to the copolymer according to the E1cB mechanism; Figure S5: 1H NMR (D2O, 600MHz) spectrum of (a) mPEG-hyd-aPHB micelles, (b) mPEG-hyd-aPHB micelles using water suppression technique; (c) mPEG-b-aPHB micelles and (d) mPEG-b-aPHB micelles (water suppression); Figure S6: CMC of (a) mPEG-hyd-aPHB and (b) mPEG-b-aPHB; Figure S7: Typical graphs of Annexin V/PI double staining apoptosis assay; Figure S8: Image processing method for a fluorescence intensity measurements, based on the Figure 12.
