# Supplementary material for: Shell-Sheddable Micelles Based on Poly(ethylene glycol)-hydrazone-poly[R,S]-3-hydroxybutyrate Copolymer Loaded with 8-Hydroxyquinoline Glycoconjugates as a Dual Tumor-Targeting Drug Delivery System
Source: Pharmaceutics. 2022 Jan 26;14(2):290. doi: 10.3390/pharmaceutics14020290 (PMC8877687; doi:10.3390/pharmaceutics14020290)
Supplement: Supplementary file 1 [file pharmaceutics-14-00290-s001.zip › pharmaceutics-1554886-supplementary.pdf]

pharmaceutics

MDPI

Supplementary Materials: Shell-Sheddable Micelles Based on Poly(Ethylene Glycol)-hydrazone-Poly[R,S]-3-Hydroxybutyrate Copolymer Loaded with 8-hydroxyquinoline Glycoconjugates as a Dual Tumor-Targeting Drug Delivery System

Adrian Domiński , Monika Domińska , Magdalena Skonieczna , Gabriela Pastuch-Gawołek and Piotr Kurcok

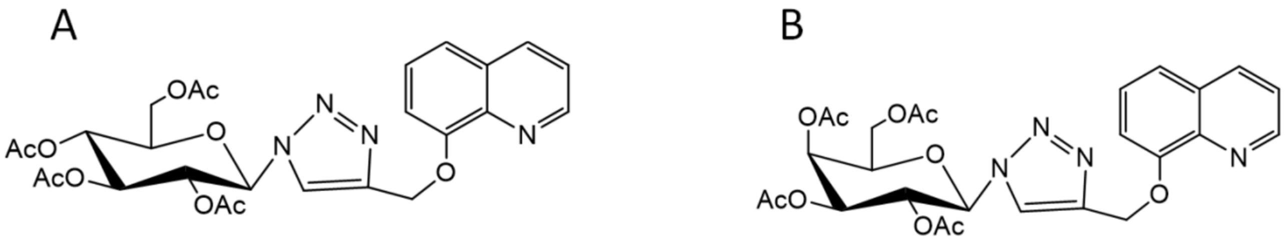

Figure S1. Structure of glycoconjugates (A) 8-((1-(2,3,4,6-tetra-*O*-acetyl-β-D-glucopyranosyl)-1H-1,2,3-triazol-4-yl)methoxy)quinoline (8HQ-glucose conjugate – 8HQ-Glu) and (B) 8-((1-(2,3,4,6-tetra-*O*-acetyl-β-D-galactopyranosyl)-1H-1,2,3-triazol-4-yl)methoxy)quinoline (8HQ-galactose conjugate – 8HQ-Gal).

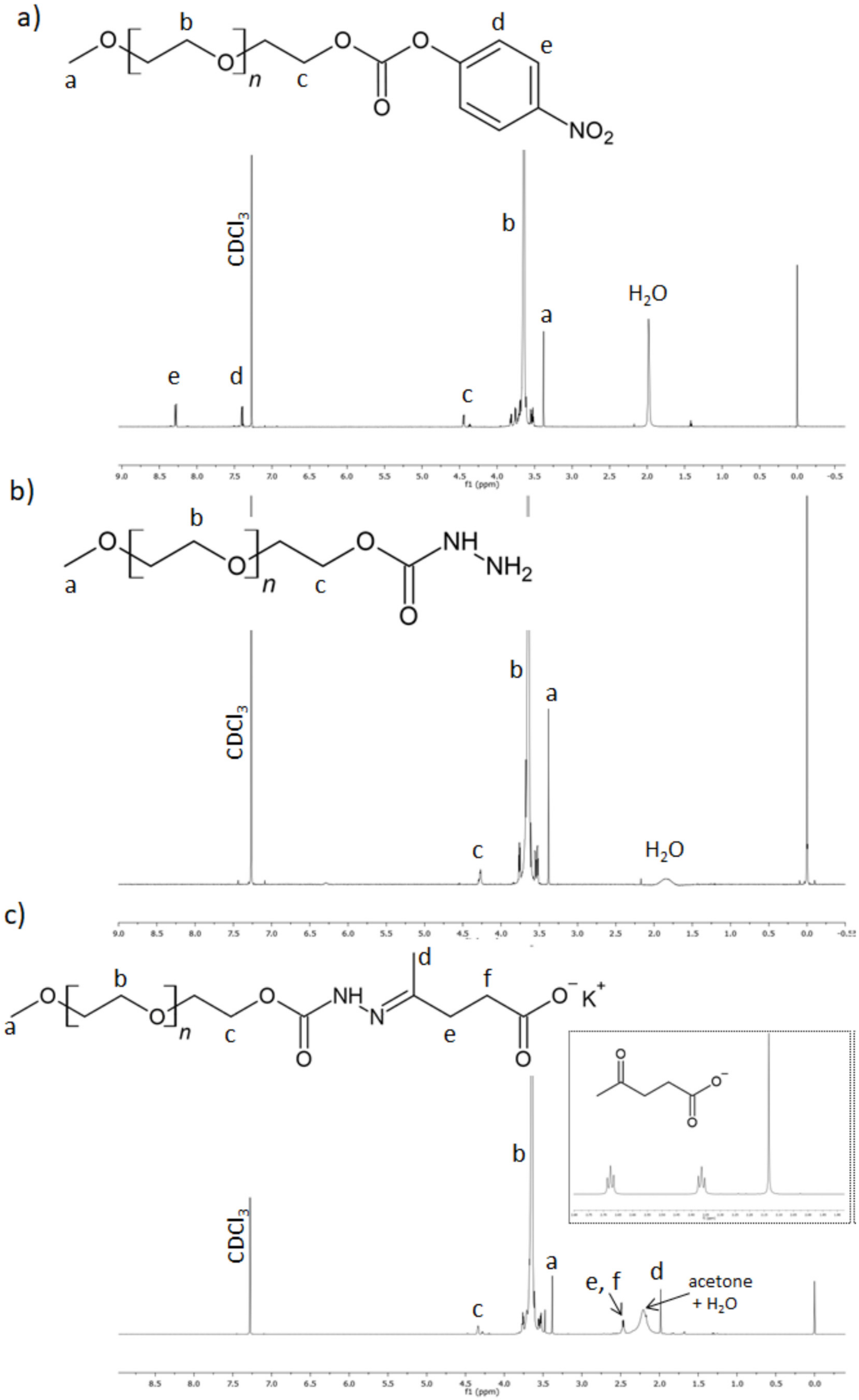

Figure S2. 1H NMR spectrum (CDCl3, 600 MHZ) of PEG-NPC, PEG-NH-NH2 and PEG-hyd-LEV.

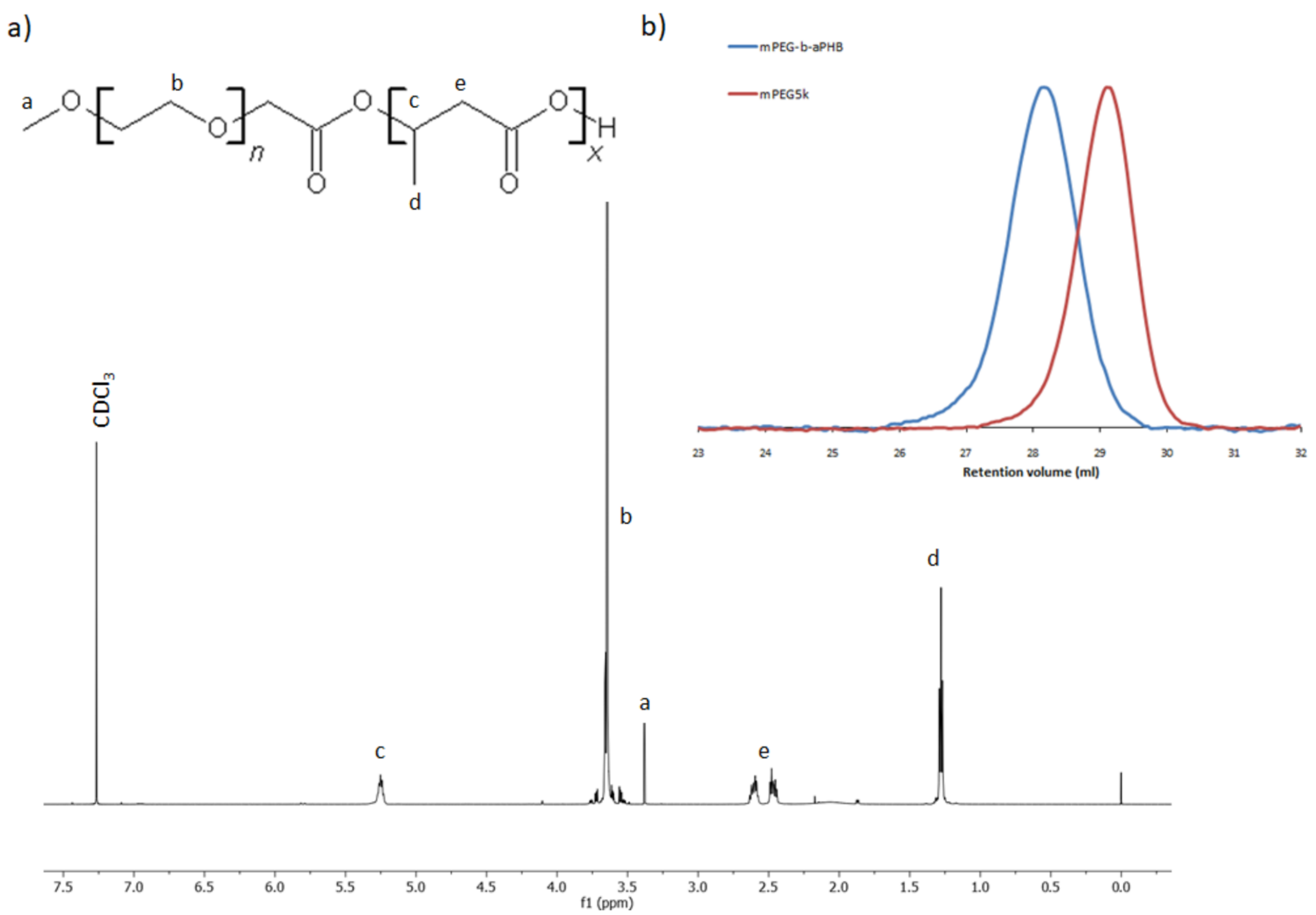

Figure S3. (a) 1H NMR spectrum (CDCl3, 600 MHz) and (b) SEC traces of mPEG-*b*-aPHB.

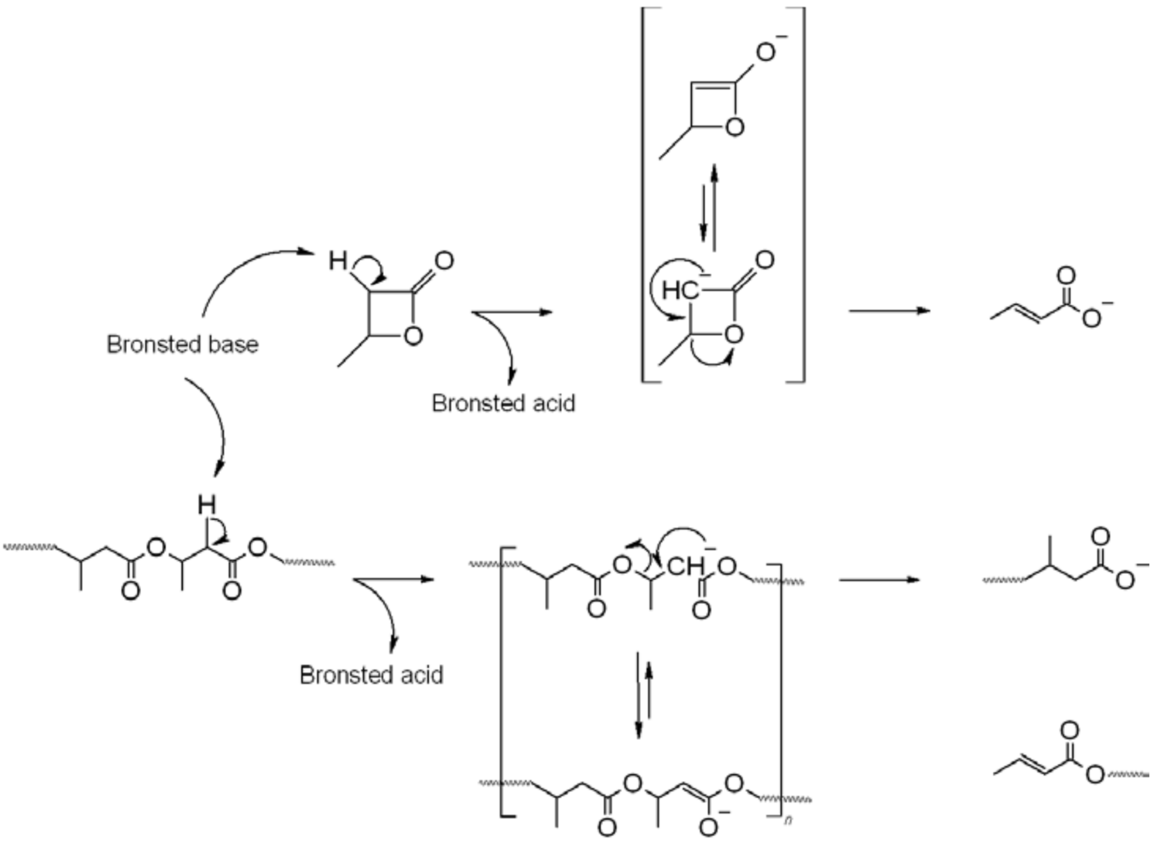

Figure S4. The possible routes of crotonate end-groups forming i.e. (i) abstraction of the acidic proton of a monomer methylene group with formation of lactone enolate, followed by elimination reaction to form a crotonate anion, which initiates further polymerization anionic ring-opening polymerization of β-butyrolactone; (ii) chain transfer to the copolymer according to the E1cB mechanism.

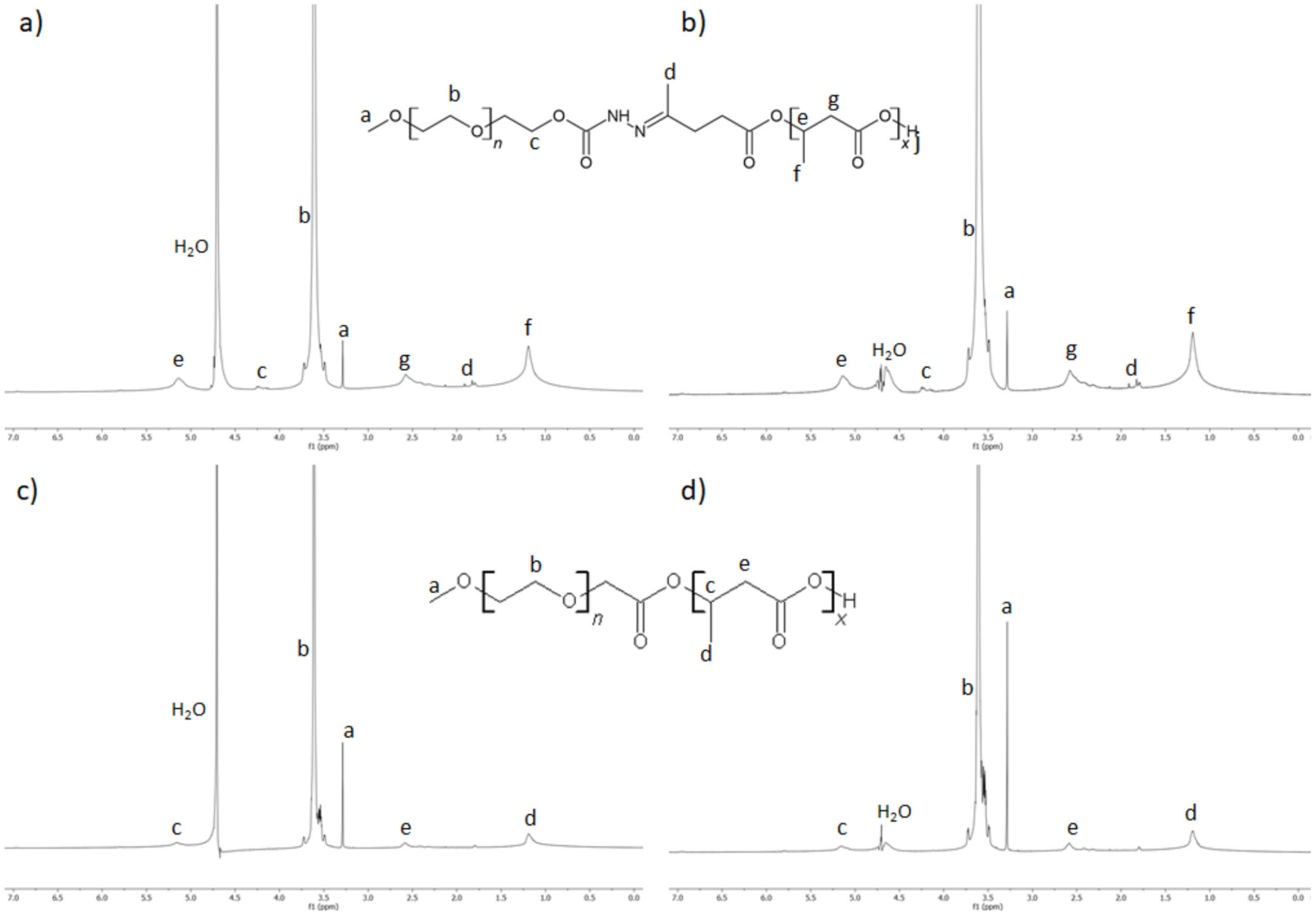

Figure S5. 1H NMR (D2O, 600MHz) spectrum of (a) mPEG-hyd-aPHB micelles, (b) mPEG-hyd-aPHB micelles using water suppression technique; (c) mPEG-*b*-aPHB micelles and (d) mPEG-*b*-aPHB micelles (water suppression).

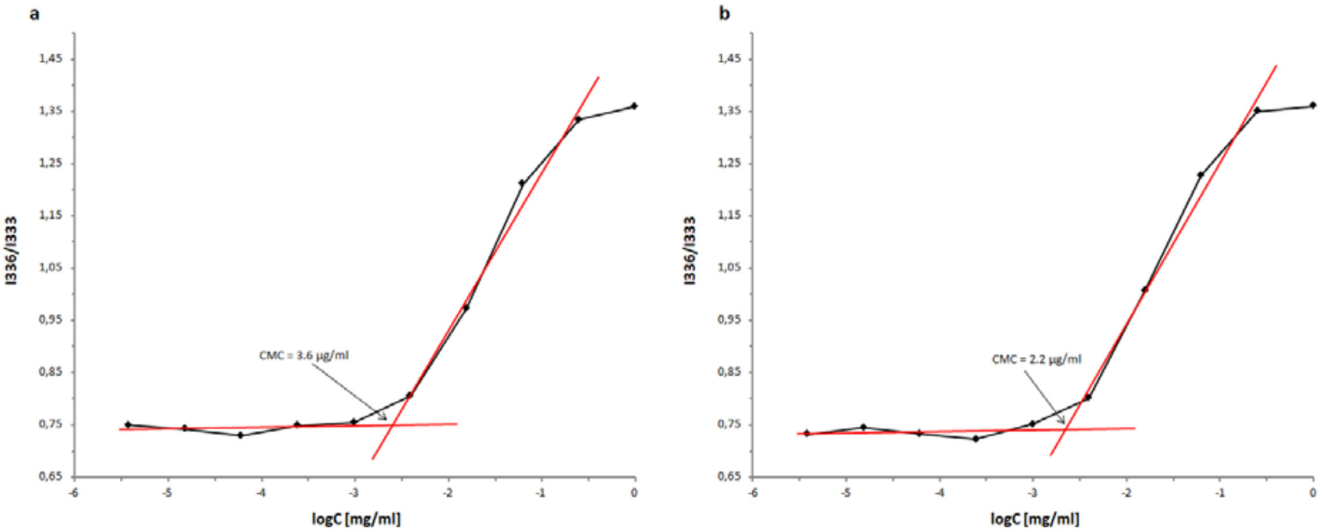

Figure S6. CMC of (a) mPEG-hyd-aPHB and (b) mPEG-b-aPHB.

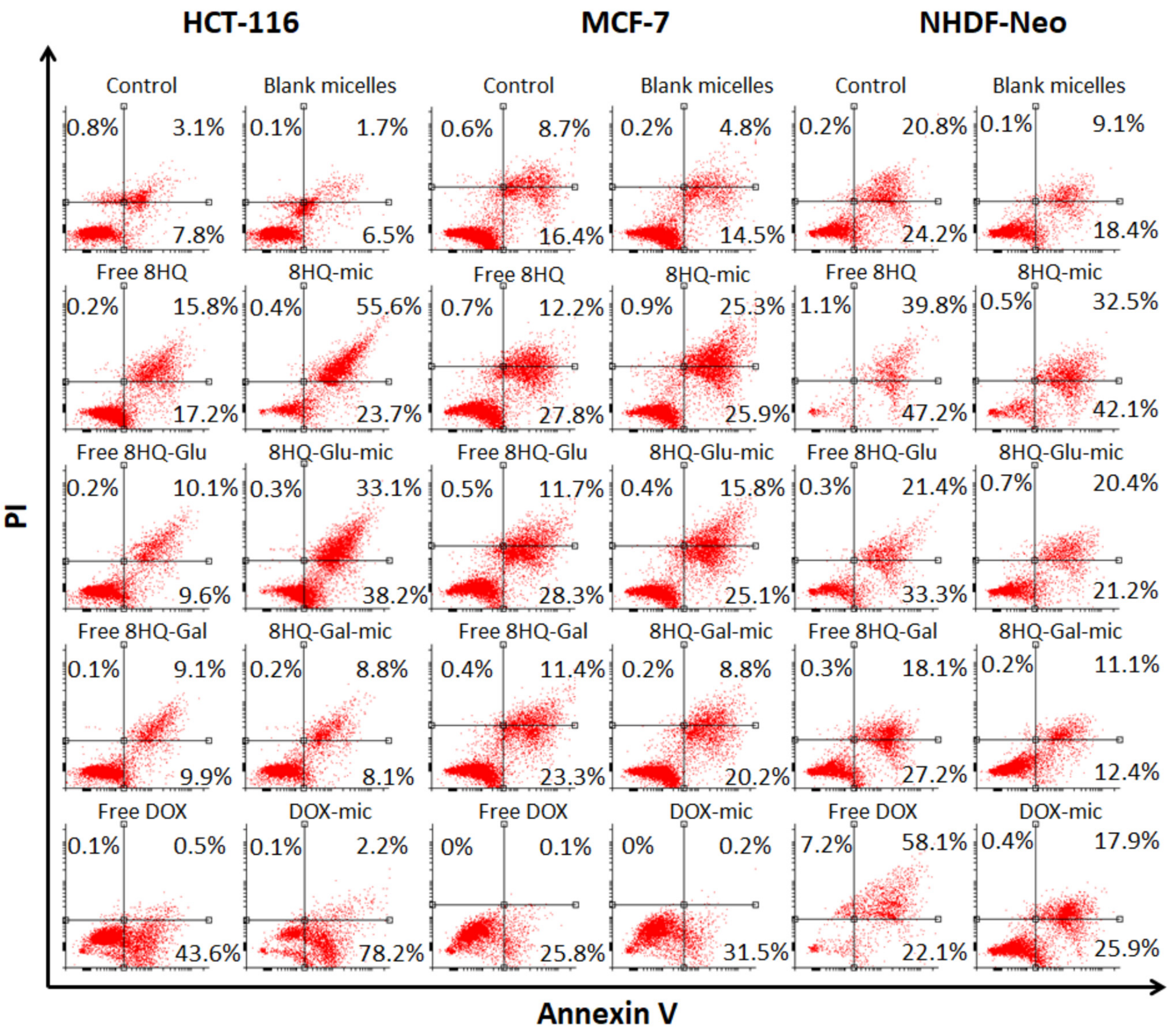

Figure S7. Typical graphs of Annexin V/PI double staining apoptosis assay.

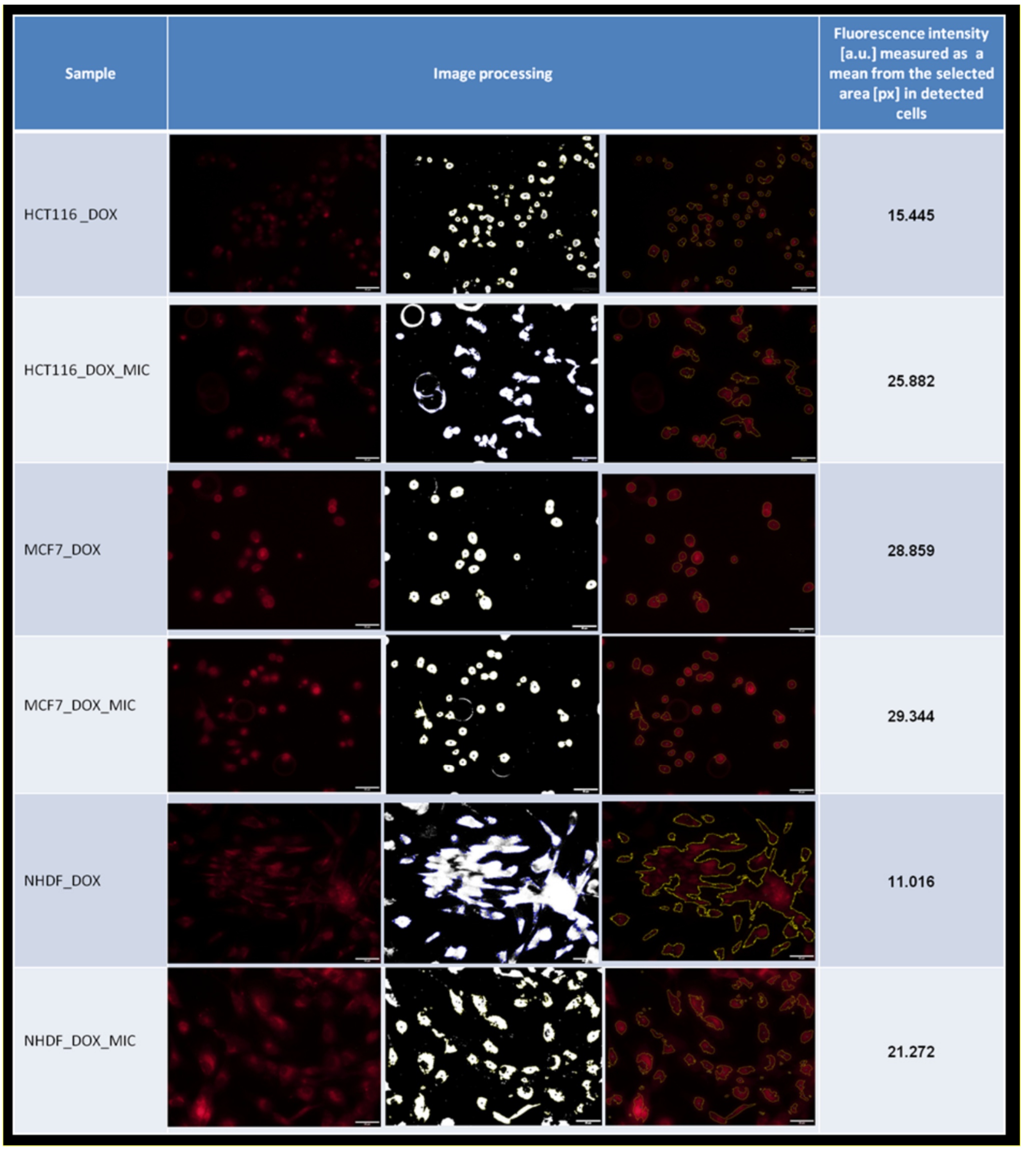

Figure S8. Image processing method for a fluorescence intensity measurements, based on the Figure 12.

Images obtained from the cells by fluorescence microscopy, acquired in a red channel (Texas Red channel) for doxorubicin (DOX) and doxorubicin-loaded micelles (DOX_MIC) were preprocessed using ImageJ 1.47v (Wayne Rasband, National Institute of Health, USA) free software. Each slide was sampled at least in triplicates and the scheme of the typical evaluation was performed. Outlines were generated based on ROI obtained from raw images (Fig. 12). In the next step, ROI was detected by the application of mean and rolling-ball filters followed by signal-to-noise thresholding. Positive cells (with red signals and fluorescence) were separated with the watershed erosion algorithm. Objects treated as cellular debris were removed from the image. In the last step of processing the fluorescence intensity [a.u.] per selected and positive cells [area; px] was calculated and presented as Mean Fluorescence Intensity.
